# 1,8-Naphthalimide-Based Multifunctional Compounds as Cu^2+^ Probes, Lysosome Staining Agents, and Non-viral Vectors

**DOI:** 10.3389/fchem.2019.00616

**Published:** 2019-09-10

**Authors:** Yong-Guang Gao, Fen-Li Liu, Suryaji Patil, Di-Jie Li, Abdul Qadir, Xiao Lin, Ye Tian, Yu Li, Ai-Rong Qian

**Affiliations:** ^1^Lab for Bone Metabolism, Key Lab for Space Biosciences and Biotechnology, School of Life Sciences, Northwestern Polytechnical University, Xi'an, China; ^2^Research Center for Special Medicine and Health Systems Engineering, School of Life Sciences, Northwestern Polytechnical University, Xi'an, China; ^3^NPU-UAB Joint Laboratory for Bone Metabolism, School of Life Sciences, Northwestern Polytechnical University, Xi'an, China

**Keywords:** 1,8-naphthalimide, Cu^2+^, lysosome, non-viral vectors, RNA delivery

## Abstract

A series of multifunctional compounds (MFCs) **1a**–**1d** based on 1,8-naphthalimide moiety were designed and synthesized. Due to the good fluorescence property and nucleic acid binding ability of 1,8-naphthalimide, these MFCs were applied in Cu^2+^ ion recognition, lysosome staining as well as RNA delivery. It was found that these MFCs exhibited highly selective fluorescence turn-off for Cu^2+^ in aqueous solution. The fluorescence emission of **1a−1d** was quenched by a factor of 116-, 20-, 12-, and 14-fold in the presence of Cu^2+^ ions, respectively. Most importantly, **1a**-Cu and **1b**-Cu could be used as imaging reagents for detection of lysosome in live human cervical cancer cells (HeLa) using fluorescence microscopy. Furthermore, in order to evaluate the RNA delivery ability of **1a−1d**, cellular uptake experiments were performed in HeLa, HepG2, U2Os, and MC3T3-E1 cell lines. The results showed that all the materials could deliver Cy5-labled RNA into the targeted cells. Among them, compound **1d** modified with long hydrophobic chain exhibited the best RNA delivery efficiency in the four tested cell lines, and the performance was far better than lipofectamine 2000 and 25 kDa PEI, indicating the potential application in non-viral vectors.

## Introduction

In recent years, considerable efforts have been paid to develop 1,8-naphthalimide derivatives as fluorescent probes, fluorescent dyes, gene vectors, and anticancer agents (Xu, 2008; Duke et al., 2010; Banerjee et al., 2014; Gao et al., 2018; Xie et al., 2018). 1,8-Naphthalimide-based fluorescence probes have been widely used for sensing cations (Cu^2+^, Zn^2+^, Hg^2+^, Ag^+^, and Pb^2+^) (Grabchev et al., 2003; Bojinov et al., 2009; Aderinto and Imhanria, 2018), anions (F^−^, CN^−^, AcO^−^, and ${\text{PO}}_{4}^{3-}$) (Jun Feng et al., 2011; Ren et al., 2011; Hao et al., 2018), and biomolecules (ATP, ADP, amino acid, and protein) (Huo et al., 2018; Seraj et al., 2018; Shahid et al., 2018). Many excellent examples of 1,8-naphthalimide-based probes have been reported, and some of them have been successfully applied in live-cell imaging research (Dai et al., 2018; Zhu et al., 2018). Furthermore, it is well-known that development of safe and efficient gene vectors is important to gene therapy (Behr, 1993; Verma et al., 1997; Niidome and Huang, 2002). Organic functional molecule as a new type of non-viral vector has received more and more attention because of its easy preparation, low immunogenicity, and good biodegradability (Seow and Yang, 2009; Pan et al., 2011; Hao et al., 2014). 1,8-naphthalimide-based functional molecules not only exhibit high transfection efficiency but also can be applied in real-time fluorescence tracking, which makes it possible to study the mechanism of gene delivery (Gao et al., 2015). It is thus no surprise that the 1,8-naphthalimide structure has made rapid development in applications for non-viral vectors, fluorescence probes, and anticancer agents in recent years.

The UV-visible absorption and fluorescence emission spectra of 1,8-naphthalimide are very sensitive to substitution in the aromatic ring. Therefore, good optical and photophysical properties can be easily achieved through structure modification (Duke et al., 2010). For example, if the 4-position of naphthalimide was substituted by the electron donating amine group, it would emit green fluorescence due to a “push-pull”-based internal charge transfer (ICT) caused by the electron donating amine and the electron withdrawing imide. In contrast, if the electron donating group such as Br moiety was exploited at the 4-position, almost no fluorescence emit was found. Therefore, different fluorescence spectra from blue to green can be achieved by altering the 4-position substituent group of naphthalimide (Bojinov et al., 2009), which allowed us easily to design the 1,8-naphthalimide derivatives that we needed. Therefore, there is huge potential to develop 1,8-naphthalimide derivatives as multifunctional compounds.

We have designed serials of 1,8-naphthalimide derivatives in recent years (Gao et al., 2016a,b, 2018). Some were applied in non-viral gene vectors for DNA delivery, and some were applied in fluorescence probes for ion recognition. However, a 1,8-naphthalimide derivative simultaneously serving as a gene vector as well as fluorescence probe has not been investigated so far. Furthermore, as a non-viral gene vector, we mainly focused on DNA delivery. However, RNA delivery mediated by these materials has never been investigated.

**Figure 1:**
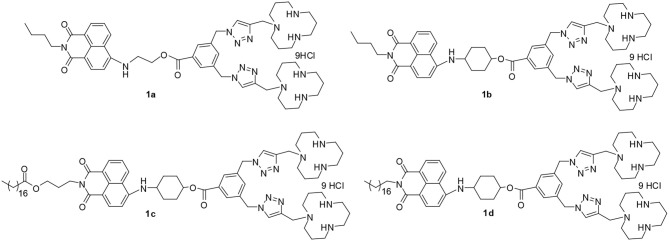
The structure of 1,8-naphthalimide derivatives.

Recently, we synthesized long hydrophobic chain modified 1,8-naphthalimide derivatives **1c** and **1d** (Figure 1), and their DNA delivery ability was investigated (Gao et al., 2016b). In this study, they will be used as fluorescence probes for recognition of Cu^2+^ and non-viral vectors for RNA delivery. In order to study the effect of the molecular structure on performance, we synthesized two other short hydrophobic chain modified 1,8-naphthalimide derivatives **1a** and **1b** (Figure 1). The performance of these four multifunctional compounds on recognition of Cu^2+^ ions and gene delivery was systematically investigated. Furthermore, the complexes of **1a**-Cu and **1b**-Cu were also investigated as lysosome probes. The results showed that the structure of these materials has significant impact on both probe performance and RNA delivery ability, which may give us clues for further design of high-performance multifunctional compounds.

## Materials and Methods

### Materials and Instruments

The agents used for reaction such as dichloromethane (DCM), triethylamine (TEA), and *N, N*-dimethylformamide (DMF) were purified by distillation before use. The solvents used for purification such as methanol (MeOH), petroleum ether (PE), and ethyl acetate (EA) were directly used without any purification. 3,5-bis(azidomethyl)benzoic acid, 1-hydroxybenzotriazole hydrate (HOBt), copper sulfate (CuSO_4_), triethylamine (TEA), 1-ethyl-3-(3-dimethylaminopropyl)carbodiimide hydrochloride (EDCI), and sodium ascorbate (Vc-Na) were purchased from Beijing Ouhe Technology Co. Ltd. (Beijing, China). Different alkyl chains modified 1,8-naphthalimide derivatives **2a**–**2b**, ditert-butyl 9-(prop-2-ynyl)-1,5,9-triazacyclododecane-1,5-dicarboxylate **5**, and the final compounds **1c** and **1d** were prepared according to our previous report (Gao et al., 2016b). Cy5-labeled RNA oligomer 5′-UUCUCCGAACGUGUCACGUTT-3′-(Cy5-RNA) was purchased from Invitrogen. 3-(4,5-dimethylthiazol-2-yl)-2,5-diphenyltetrazolium bromide (MTT) was purchased from Solarbio company (Beijing, China).

^1^H NMR and ^13^C NMR spectra were collected using a Bruker Avance spectrometer. Mass spectra were obtained on a Waters Quattro Mocro spectrometer and high-resolution mass spectra were acquired on a Waters LCT Premier XE spectrometer. The infrared spectra were acquired on a Nicolet 380 spectrometer. Fluorescence spectra were measured on a Hitachi F-4500 fluorescence spectrophotometer. Hydrodynamic diameters and zeta potentials were collected using a Nano-ZS 3600 zetaplus particle size and zeta potential analyzer. Cell images were observed by a Leica DMI8 Inverted Fluorescence Microscope (Wetzlar, Germany).

### Synthesis of Multifunctional Compounds 1a−1b

#### Synthesis of Compound 4a/4b

3,5-Bis(azidomethyl)benzoic acid (0.81 mmol) was dissolved in SOCl_2_ (2 mL). After stirring for 2 h at 70°C, the surplus SOCl_2_ was evaporated under reduced pressure. The residue was dissolved in dichloromethane (5 mL) and TEA (1.34 mmol). Compound **2a**/**2b** (0.67 mmol) was added and stirred for 24 h at room temperature. Water (5 mL) was added and the mixture was extracted with DCM (2 × 15 mL). The organic phase was dried (Na_2_SO_4_), filtered, and the solvent was evaporated under reduced pressure. The crude material was purified by column chromatography on silica gel (PE/EA = 4/1) to give a yellow product.

**4a**, 81%; ^1^H NMR (400 MHz, CDCl_3_) δ 8.59 (d, *J* = 7.3 Hz, 1H), 8.48 (d, *J* = 8.3 Hz, 1H), 8.13 (d, *J* = 8.3 Hz, 1H), 7.99 (s, 2H), 7.66 (t, *J* = 7.9 Hz, 1H), 7.51 (s, 1H), 6.77 (d, *J* = 8.4 Hz, 1H), 6.02 (s, 1H), 4.92 – 4.79 (m, 2H), 4.44 (s, 4H), 4.20 – 4.12 (m, 2H), 3.84 (dd, *J* = 9.9, 4.9 Hz, 2H), 1.70 (t, *J* = 6.7 Hz, 3H), 1.46 – 1.41 (m, 3H), 0.97 (t, *J* = 7.3 Hz, 4H); ^13^C NMR (101 MHz, CDCl_3_) δ 166.77, 164.59, 164.07, 149.00, 137.03, 134.15, 132.37, 132.23, 131.12, 130.92, 130.60, 129.66, 128.92, 126.10, 125.02, 123.14, 120.38, 111.05, 104.13, 65.57, 63.59, 53.95, 43.63, 39.99, 30.55, 30.30, 20.42, 19.17, 13.88, 13.72; IR (KBr, cm^−1^): 3360.24, 2956.33, 2872.29, 2107.83, 1720.18, 1682.23, 1638.86, 1576.77, 1576.51, 1381.73, 1207.83, 1115.66, 771.39; ESI-MS: m/z = 527.5 ([M+H]^+^).

**4b**, 63%; ^1^H NMR (400 MHz, CDCl_3_) δ 8.60 (d, *J* = 6.8 Hz, 1H), 8.48 (d, *J* = 8.4 Hz, 1H), 8.08 (d, *J* = 8.0 Hz, 1H), 7.98 (s, 2H), 7.67 – 7.62 (m, 1H), 7.51 (s, 1H), 6.78 (d, *J* = 8.6 Hz, 1H), 5.12 – 5.07 (m, 2H), 4.47 (s, 4H), 4.17 (t, *J* = 7.6 Hz, 2H), 3.75 – 3.72 (m, 1H), 2.39 (d, *J* = 11.4 Hz, 2H), 2.29 (d, *J* = 10.7 Hz, 2H), 1.85 – 1.60 (m, 6H), 1.47 – 1.41 (m, 2H), 0.97 (t, *J* = 7.4 Hz, 3H); ^13^C NMR (101 MHz, CDCl_3_) δ 165.14, 164.60, 164.06, 148.21, 136.83, 134.25, 131.96, 131.70, 131.14, 129.95, 128.95, 125.78, 124.74, 123.28, 120.28, 110.50, 104.64, 72.81, 54.10, 50.8, 39.99, 30.32, 30.25, 29.99, 20.43, 13.89; IR (KBr, cm^−1^): 3409.04, 2959.04, 2096.99, 1682.23, 1644.28, 1581.93, 1386.75, 1365.06, 1221.39, 1107.53, 774.10; ESI-MS: m/z = 580.6 ([M+H]^+^).

#### Synthesis of Compound 6a/6b

To a solution of **4a**/**4b** (0.47 mmol) and compound **5** (0.96 mmol) in THF/H_2_O (10 mL/5 mL), copper sulfate (0.047 mmol), and Vc-Na (0.1 mmol) were added. The mixture was stirred overnight at room temperature. The solvent was removed under reduced pressure. Water (10 mL) was added and the mixture was extracted with DCM (2 × 10 mL). The combined organic layer was washed with saturated brine, dried over Na_2_SO_4_, filtered, and the solvent was evaporated under reduced pressure. The crude material was purified by column chromatography on silica gel (CH_2_Cl_2_/MeOH = 20/1) to give a yellow product.

**6a**, 77%; ^1^H NMR (400 MHz, CDCl_3_) δ 8.57 (d, *J* = 7.3 Hz, 1H), 8.45 (d, *J* = 8.4 Hz, 1H), 8.21 (d, *J* = 8.4 Hz, 1H), 7.87 (s, 2H), 7.65 (t, *J* = 7.9 Hz, 1H), 7.39 (s, 3H), 6.76 (d, *J* = 8.5 Hz, 1H), 6.26 (s, 1H), 5.54 (s, 4H), 4.73 (t, *J* = 4.9 Hz, 2H), 4.19 – 4.10 (m, 2H), 3.89 – 3.66 (m, 6H), 3.45 – 3.18 (m, 16H), 2.43 (s, 8H), 2.04 – 1.73 (m, 12H), 1.73 – 1.65 (m, 2H), 1.50 – 1.36 (m, 38H), 0.96 (t, *J* = 7.3 Hz, 3H); ^13^C NMR (101 MHz, CDCl_3_) δ 165.75, 164.45, 163.89, 156.22, 149.50, 144.26, 136.69, 134.08, 131.69, 131.22, 130.96, 129.60, 128.97, 126.80, 124.69, 122.78, 120.39, 110.35, 104.05, 79.24, 77.54, 77.23, 76.91, 63.62, 52.96, 49.60, 46.70, 45.32, 43.74, 42.90, 39.82, 30.22, 28.41, 27.20, 25.95, 20.32, 13.83; IR (KBr, cm^−1^): 3409.40, 2969.88, 1687.65, 1649.70, 1584.64, 1365.06, 1245.78, 1167.17, 1050.60, 779.52; ESI-MS: m/z = 1345.8 ([M+H]^+^).

**6b**, 69%; ^1^H NMR (400 MHz, CDCl_3_) δ 8.60 (d, *J* = 6.9 Hz, 1H), 8.48 (d, *J* = 8.4 Hz, 1H), 8.07 (d, *J* = 8.2 Hz, 1H), 7.92 (s, 2H), 7.64 (t, *J* = 7.9 Hz, 1H), 7.38 (d, *J* = 6.0 Hz, 3H), 6.77 (d, *J* = 8.6 Hz, 1H), 5.56 (s, 4H), 5.13 – 5.01 (m, 2H), 4.21 – 4.13 (m, 2H), 3.84 – 3.66 (m, 5H), 3.34 – 3.32 (m, 16H), 2.58 – 2.20 (m, 12H), 2.06 – 1.66 (m, 18H), 1.61 – 1.53 (m, 2H), 1.48 – 1.41 (m, 36H), 0.97 (t, *J* = 7.4 Hz, 3H); ^13^C NMR (101 MHz, CDCl_3_) δ 164.54, 163.94, 156.23, 148.45, 144.24, 136.52, 134.25, 132.19, 131.48, 131.00, 129.88, 129.06, 126.25, 124.49, 123.00, 122.55, 120.29, 110.08, 104.52, 79.23, 73.09, 53.15, 50.76, 49.63, 46.67, 45.36, 43.81, 39.86, 30.24, 30.04, 29.93, 28.43, 28.22, 27.18, 26.02, 20.34, 13.83; IR (KBr, cm^−1^): 3425.30, 2972.59, 2931.93, 1684.94, 1649.99, 1581.93, 1386.75, 1365.06, 1221.39, 1164.46, 774.10; ESI-MS: m/z = 1399.9 ([M+H]^+^).

#### Synthesis of Compound 1a/1b

Compound **6a**/**6b** (0.19 mmol) was added to a solution of hydrogen chloride in ethyl acetate (10 mL) and the mixture was stirred for 30 min at room temperature. The resulting suspension was filtrated, and the solid was washed with ethyl acetate and dried in vacuum at 60°C for 24 h.

**1a**, 77%; ^1^H NMR (400 MHz, D_2_O) δ 7.56 (s, 2H), 7.21 – 7.15 (m, 2H), 7.01 (s, 2H), 6.93 (s, 3H), 6.81 (s, 1H), 6.55 (s, 1H), 5.59 (s, 1H), 4.91 (s, 5H), 3.87 (s, 4H), 3.66 (s, 4H), 3.06 (s, 4H), 2.94 – 2.72 (m, 16H), 2.61 (s, 8H), 1.74 (s, 4H), 1.60 (s, 8H), 0.79 (s, 2H), 0.66 (s, 2H), 0.30 (s, 3H); ^13^C NMR (101 MHz, CDCl_3_) δ 169.05, 167.01, 166.05, 153.01, 141.05, 138.25, 136.30, 134.91, 133.17, 132.75, 131.42, 130.54, 130.20, 129.07, 126.50, 122.40, 121.29, 109.20, 106.49, 66.98, 59.81, 55.31, 51.22, 49.50, 45.44, 44.14, 43.58, 42.24, 31.97, 22.32, 22.17, 21.41, 19.20, 15.55; IR (KBr, cm^−1^): 3425.30, 2959.04, 1714.76, 1636.14, 1581.93, 1386.75, 1362.35, 1218.67, 1121.08, 776.81; HR-MS: m/z = 945.5948 ([M+H]^+^)

**1b**, 82%; ^1^H NMR (400 MHz, D_2_O) δ 8.02 (s, 2H), 7.80 (s, 1H), 7.69 (s, 1H), 7.58 – 7.54 (m, 3H), 7.20 (s, 1H), 7.07 (s, 1H), 6.07 (s, 1H), 5.40 (s, 4H), 4.02 (s, 4H), 3.52 (s, 2H), 3.22 – 2.93 (m, 26H), 2.15 (s, 4H), 1.98 (s, 12H), 1.46 – 1.01 (m, 8H), 0.63 (s, 3H); ^13^C NMR (101 MHz, D_2_O) δ 165.60, 164.37, 163.41, 149.30, 137.14, 135.82, 133.96, 132.28, 131.00, 128.91, 128.47, 127.44, 127.44, 123.81, 120.31, 119.07, 106.91, 103.84, 73.86, 52.99, 50.52, 47.87, 42.32, 41.26, 29.64, 29.07, 19.94, 19.83, 18.29, 13.27; IR (KBr, cm^−1^): 3422.59, 2956.33, 2858.73, 1684.94, 1644.28, 1581.93, 1384.04, 1302.71, 1221.39, 1110.24, 779.52; HR-MS: m/z = 999.6400 ([M+H]^+^).

### Measurement Procedure

#### UV and Fluorescent Spectral Measurements

The stock solutions of multifunctional organic compounds (MFCs) **1a**–**1d** (1 mM) and metal ions (10 mM) were prepared in tri-distilled water and stored at 4°C for use. The fluorescence emission and ultraviolet absorption experiments were carried out in Tris-HCl buffer (1 mM, pH 7.2). Test solution was prepared by placing MFCs **1a**–**1e**, Tris-HCl buffer solution and an appropriate volume of each analyte into 3 mL of cuvette. After equilibration for 2 min, an ultraviolet absorption and fluorescence emission spectrum was recorded at 25°C.

#### Cell Culture and Fluorescence Imaging

Cell culture: HeLa cells (a human cervical carcinoma cell line) were cultured with DMEM containing 100 units/mL penicillin sulfate and streptomycin, medium supplemented with 10% fetal bovine serum at 37°C under 5% CO_2_ for 24 h.

Cell imaging (Cu^2+^ recognition): cells were seeded in glass bottom cell culture dish (8 × 10^4^ cells), and incubated with 20 μM of MFCs **1a**–**1d** for 0.5 h. After incubation, the cells were washed with PBS 4 times and imaged under a fluorescence microscope. Then, 10 equivalents of Cu(ClO_4_)_2_ was added to the cells, and fluorescence images were taken one time per 15 min under a Leica DMI8 Inverted Fluorescence Microscope. Fluorescence images were obtained using a 10 × objective lens. Cell images were processed and analyzed using the Image-Pro Plus software. Three repeats were conducted for each sample.

Lysosome imaging: The cells were seeded in 24-well plates at 8 × 10^4^ cells per well and grew for 24 h. After removing the medium, **1a (1b)**-Cu complexes (20 μM) were added and incubated for 30 min in DMEM medium. Then the medium was replaced with fresh medium containing Lyso-Tracker Red (10 μM) and incubated for another 30 min. The samples were then imaged by an Inverted Fluorescence Microscope after washing with cell culture medium. Lyso-Tracker Red images were observed in the red channel and **1a (1b)**-Cu images were observed in the green channel with a 20 × objective lens. Cell images were processed and analyzed using the Image-Pro Plus software. Three repeats were conducted for each sample.

#### Dynamic Light Scattering (DLS)

The complexes of MFCs **1a**–**1d** with RNA were prepared by adding 0.7 μL of siRNA (264 μg/mL, 5′-UUCUCCGAACGUGUCACGUTT-3′) to the appropriate volume of the stock solutions of **1a**–**1d**. Then the complex solution was vortexed for 30 s and then diluted up to 0.5 mL by tri-distilled water. The zeta potentials and the hydrodynamic diameters were measured using a Nano-ZS 3600 zetaplus particle size and zeta potential analyzer.

#### Cellular Uptake Experiment

The cellular uptake of the complexes of MFCs **1a**–**1d** with Cy5-labeled siRNA was obtained by fluorescence microscope. HeLa, HepG2, and U2Os cells were cultured with DMEM medium, and MC3T3-E1 cells were cultured with α-MEM medium. The cells were seeded in 24-well plates at 8 × 10^4^ cells per well and grew for 24 h. After washing with DMEM, the cells were incubated with freshly prepared complexes of MFCs **1a**–**1d** with Cy5-RNA (9 μg/mL) and the controls (500 μL). After 4 h incubation, the cells were washed 6 times with PBS buffer. Cells were observed by a Leica DMI8 Inverted Fluorescence Microscope, Cy5-labeled RNA images were observed in the red channel, and MFCs images were observed in the green channel with a 10 × objective lens. Cell images were processed and analyzed using the Image-Pro Plus software. Three repeats were conducted for each sample.

#### Cytotoxicity Experiment

The cytotoxicity of **1a**–**1d**/RNA complexes toward HeLa cell lines was tested by MTT assays. The cells were seeded into 96-well plates at densities of 5 × 10^3^ cells in 100 μL DMEM medium. After culture for 24 h, the complexes (**1a**–**1d**/RNA) were added to cells at various concentrations (10, 15, 20, and 25 μM). After incubation for 4 h, the medium was replaced with 200 μL of fresh medium containing 10% FBS, and cells were cultured for another 48 h. Subsequently, 20 μL of MTT (5 mg/mL) solution in PBS was added to each well for an additional 4 h incubation. The MTT medium was replaced by 200 μL of DMSO. The absorbance was measured using a microplate spectrophotometer at a wavelength of 490 nm. The cells treated without any complexes were used as controls. The relative viability of the cells was calculated based on the data of five parallel tests by comparing to the controls.

## Results and Discussion

### Synthesis of Multifunctional Compounds 1a−1d

**Scheme 1:**
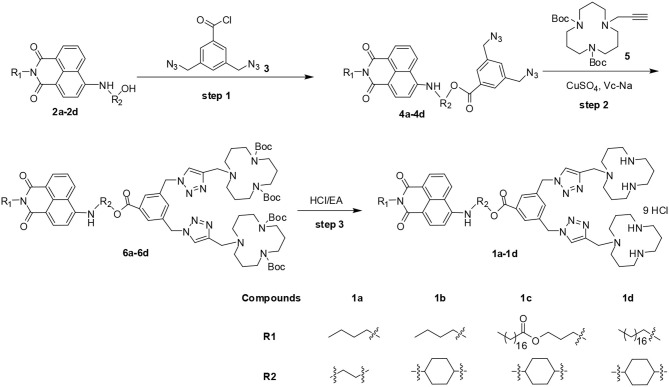
Synthetic routes of multifunctional compounds **1a**–**1d**.

1,8-naphthalimide-based MTCs **1c** and **1d** were prepared according to our previous report (Gao et al., 2016b), and two other new compounds **1a** and **1b** were synthesized based on a similar method. As shown in Scheme 1, compound **4** was synthesized through acylation of **2** with 3,5-bis(azidomethyl)benzoyl chloride **3**. Further reaction of **4** with propargyl [12]-aneN_3_ **5** catalyzed by CuSO_4_ and sodium ascorbate (Vc-Na) gave intermediate **6**. The final compounds were obtained through de-protection of **6** under acidic condition. All the new compounds were fully characterized by ^1^H NMR, ^13^C NMR, IR, and MS.

### Spectroscopic Properties of 1a−1d

**Figure 2:**
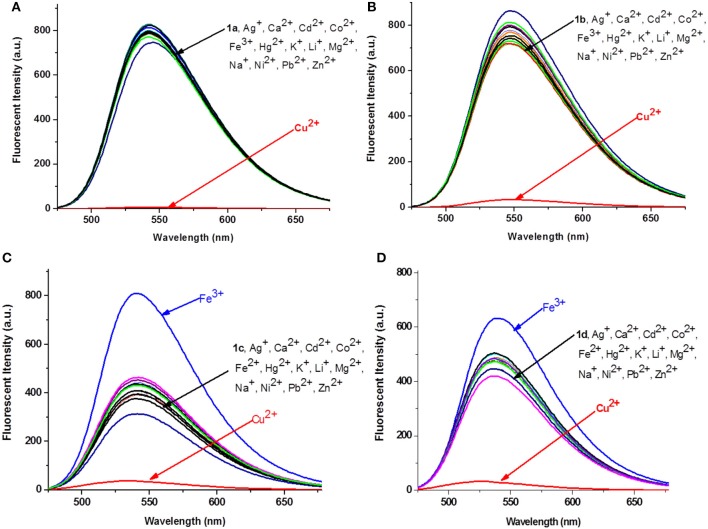
Fluorescence changes **(A–D)** of **1a−1d** (1 × 10^−5^ M, Tris-HCl buffer, 1 mM) upon the addition of 5 equivalents of various metal ions.

The absorption and fluorescence spectra of **1a−1d** were measured in water-Tris-HCl buffer (1 mM, pH = 7.2). As shown in Figure S1A, all the compounds exhibited three similar absorption peaks in the range of 250–525 nm. The broadest absorption peak ranging from 350 to 525 nm should be produced by 1,8-naphthalimide unit. The fluorescence spectra of **1a−1d** showed that there were no obvious changes on the maximum emission wavelength, which appeared at 541, 546, 540, and 536 nm, respectively (Figure S1B). Subsequently, the fluorescence spectra changes of **1a−1d** were measured upon addition of 5 equivalents of different metal ions (Ag^+^, Ca^2+^, Cd^2+^, Co^2+^, Fe^3+^, Hg^2+^, K^+^, Li^+^, Mg^2+^, Na^+^, Ni^2+^, Pb^2+^, Zn^2+^, and Cu^2+^) in Tris-HCl buffer. As shown in Figure 2, the fluorescence intensities of **1a**–**1d** decreased significantly after addition of Cu^2+^, and the fluorescence was reduced about 116-, 20-, 12-, and 14-fold, respectively. However, no obvious fluorescence decrease was observed for other metal ions. Interestingly, Fe^3+^ could produce obvious enhancement of the fluorescence intensity of **1c** (~1-fold). However, it had no obvious influence on fluorescence of other probes such as **1a**, **1b**, and **1d**. The fluorescence enhancement phenomenon should be ascribed to the special structure of **1c**. Two carbonyl groups existed in 1,8-naphthalimide, and the long hydrophobic chain might be coordinated with Fe^3+^, which could effectively inhibit photoinduced electron transfer (PET) of 1,8-naphthalimide (Kucheryavy et al., 2011). These results could give us clues to design high selective Cu^2+^ and Fe^3+^ probes.

**Figure 3:**
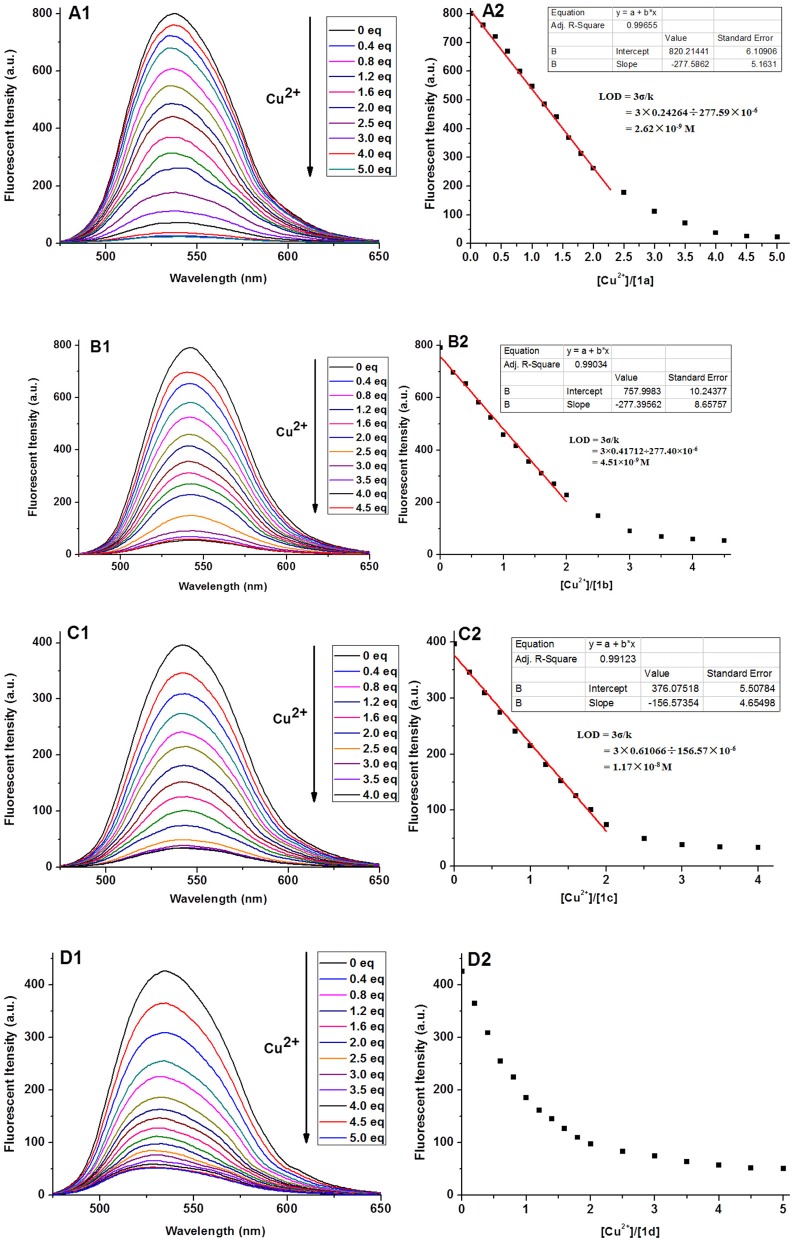
Fluorescence spectra **(A1–D1)** of **1a**–**1d** (10 μM) upon the addition of Cu^2+^ and the fluorescence intensity **(A2–D2)** as a function of the molar ratio ([Cu^2+^]/[**1**]) in aqueous solution (1 mM Tris-HCl, pH 7.2).

The fluorescence titrations of **1a**–**1d** with Cu^2+^ were performed in 1 mM Tris-HCl buffer. As shown in Figure 3, after addition of Cu^2+^, the fluorescence of **1a**–**1d** was quenched significantly, with the maximum emission at 541, 546, 540, and 536 nm, respectively. Furthermore, we found that a good linear relationship existed between Cu^2+^ concentration and fluorescent intensities of **1a**, **1b**, and **1c** (0–2.0 equiv. of Cu^2+^). According the formula (LOD = 3σ/k), the limits of detections of **1a−1c** were calculated to be 2.62 × 10^−9^, 4.51 × 10^−9^, and 1.17 × 10^−8^ M, respectively, which are greatly lower than the limit of Cu^2+^ ions in drinking water (20 μM) determined by the EPA (Zhu et al., 2012). Note that, for probe **1d**, this linear relationship was changed less obviously ranging from 0 to 2.0 equivalents of Cu^2+^, which could be explained by stating that the long alkyl chain was not good for coordination between 1,8-naphthalimide and Cu ^2+^. To confirm the stoichiometry between **1** and Cu^2+^, Job's plot analyses were carried out. As shown in Figure S2, a 1:2 binding mode was found between compound **1** and Cu^2+^, which was in agreement with the fluorescence titration analyses. The binding manner of **1** with Cu^2+^ was given referring to the above results and our previous report (Gao et al., 2016a). As shown in Figure S3, The quenching effects of **1a**–**1d** should be ascribed to the coordination of the amino group linked with 1,8-naphthalimide and the triazole-[12]aneN_3_ unit with Cu^2+^ as well as the paramagnetic effect of Cu^2+^ ions (Rorabache, 2004; Huang et al., 2014).

### Solvents Effect on Cu^2+^ Recognition

**Figure 4:**
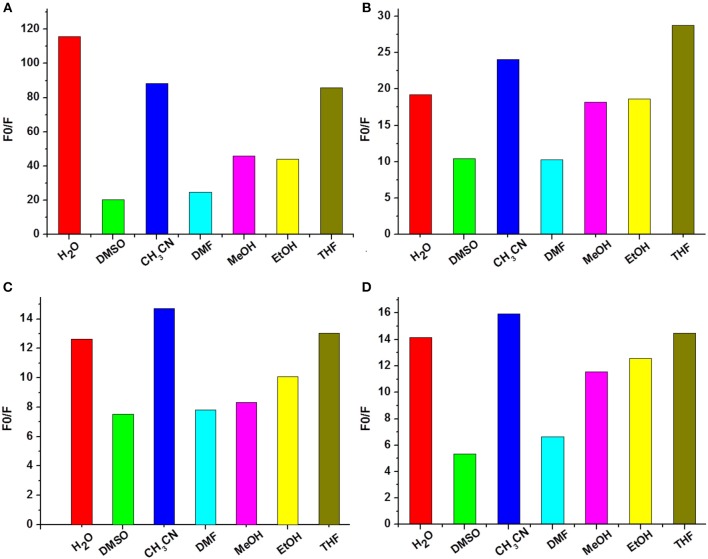
Fluorescence changes **(A–D)** of **1a**–**1d** (10 μM) upon the addition of Cu^2+^ in different solvents (1 mM Tris-HCl, pH 7.2).

As high performance Cu^2+^ sensors, they should have the ability to resist interference from complex environments. Therefore, fluorescence changes of compounds **1a**–**1d** were measured upon addition of 5 equivalents of Cu^2+^ in water (H_2_O), dimethyl sulfoxide (DMSO), acetonitrile (CH_3_CN), *N, N*-dimethylformamide (DMF), methanol (MeOH), ethanol (EtOH), and tetrahydrofuran (THF) solvents. As shown in Figure 4, generally speaking, water, acetonitrile, and tetrahydrofuran were good solvents for **1a**–**1d** to recognize Cu^2+^. Especially for probe **1a** (Figure 4A), its fluorescence intensity was decreased more than 80-fold in the presence of Cu^2+^ in the above three solvents. DMSO and DMF, by contrast, gave weak Cu^2+^ recognition effect. However, the fluorescence of **1a**–**1d** still reduced more than 5-fold upon addition of Cu^2+^ in all tested solvents. These results make us believe that compounds **1a**–**1d** can be applied as fluorescence probes for recognition Cu^2+^ in different solvents, owing to their strong anti-interference capabilities.

### pH Dependence

**Figure 5:**
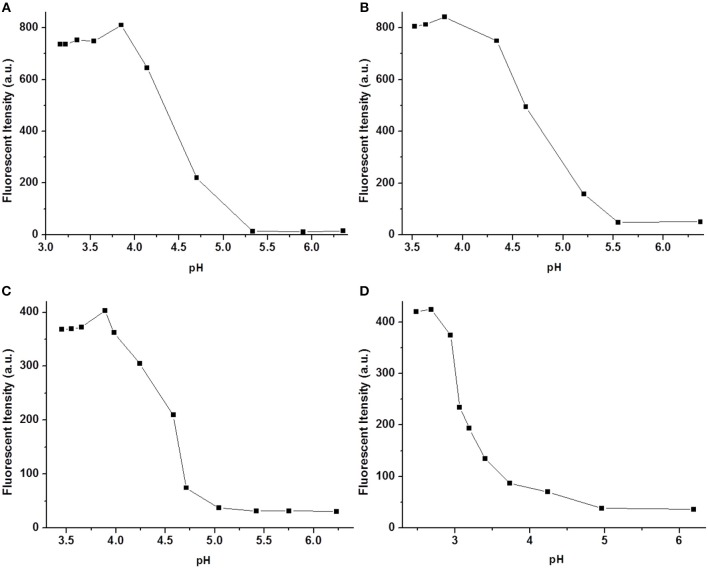
Fluorescence spectra **(A–D)** of **1a**–**1d** (10 μM) upon the addition of Cu^2+^ in aqueous solution (1 mM Tris-HCl, pH 7.2).

The pH responses of **1a−1d** were also investigated in the presence of 5 equivalents of Cu^2+^ in aqueous solution. As shown in Figure 5, the fluorescence of the **1**-Cu complex was almost thoroughly quenched at pH > 5.5. Therefore, **1a−1d** can probably be employed as useful probes for recognition of Cu^2+^ under biological conditions. Interestingly, a significant fluorescence change was observed in the pH range from 4.0 to 5.5. It is well-known that the pH value of lysosome lumen ranges from 4.0 to 6.0, while the cytoplasm is about 7.2 (Zhang et al., 2015), which inspires us to use the complexes of **1**-Cu to stain lysosome in living cells.

### Cell Imaging

**Figure 6:**
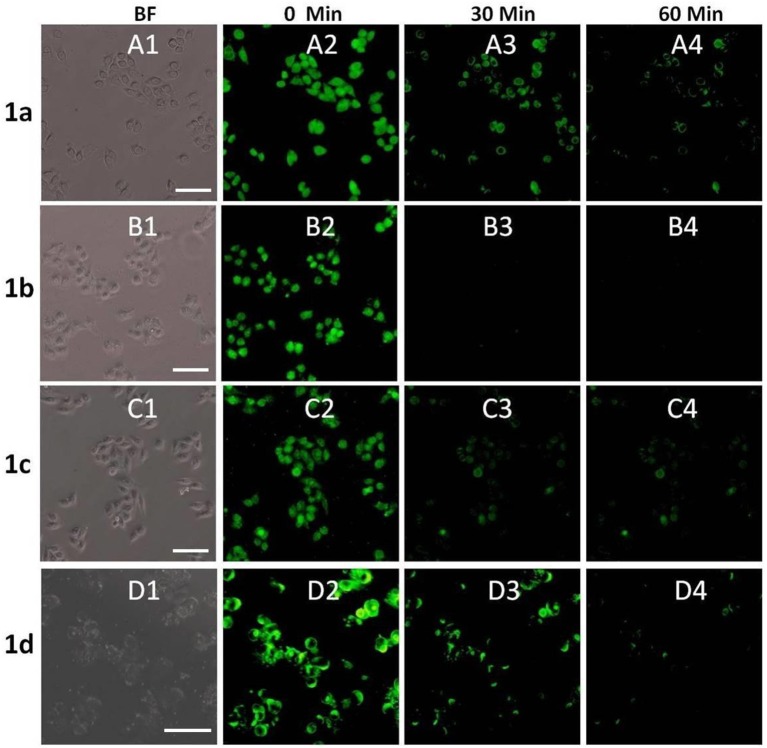
Fluorescence microscope images of HeLa cells incubated with **1a**–**1d** (20 μM) upon addition of Cu^2+^ (bright field: **A1–D1**) for 0 min **(A2–D2)**, 30 min **(A3–D3)** and 60 min **(A4–D4)**. The scale bar in the figure is 30μm.

In order to investigate the biological applications of **1a**–**1d**, the fluorescence microscopy experiment was carried out in living cells. As shown in Figures 6A1–D1, the cell retained its normal morphology after being incubated with **1a**–**1d**. All the probes can readily penetrate living HeLa cells and emit bright green fluorescence under a fluorescence microscope (Figures 6A2–D2). When 10 equivalents of Cu^2+^ were added, the fluorescence intensities of **1a**–**1d** decreased significantly (Figures 6A3–D3). After 60 min, almost no obvious fluorescence change was observed (Figures 6A4–D4). However, the fluorescence was not completely quenched, especially for **1a** and **1b**, and bright green fluorescence was still observed, which could be caused by the acidic environment of lysosomes (pH 4.0–6.0). It was also found that the cellular morphology was changed obviously after addition of compound **1d** and Cu^2+^, which might be caused by the toxicity of **1d** and free copper ions. These results further showed that the **1**-Cu complex could be used as a pH probe for lysosome imaging.

### Lysosome Imaging

**Figure 7:**
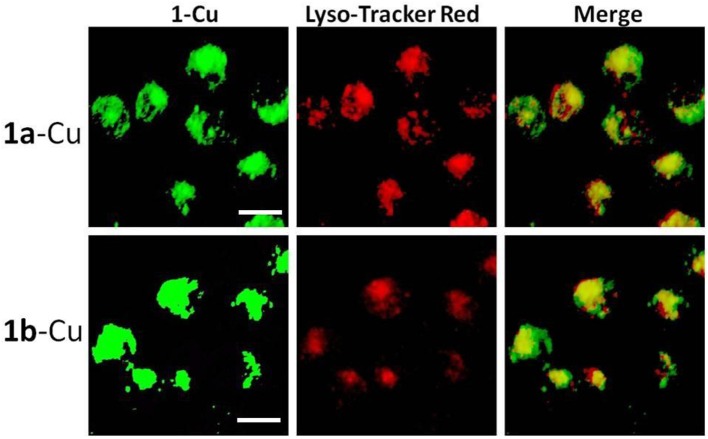
Fluorescence microscope images of HeLa cells incubated with **1a**-Cu and **1b**-Cu complexes (20 μM) for 0.5 h and Lyso-Tracker Red for 0.5 h. A representative result from three independent experiments. The scale bar in the figure is 10 μm.

It is well-known that lysosomes are one of the important organelles in eukaryotic cells. They play a key role in the degradation of macromolecules and cell components (Wong et al., 2018). The fluorescence microscopy results show that **1a**–**1d** can easily penetrate living HeLa cells, and the fluorescence of **1a**/**1b** cannot be completely quenched by Cu^2+^. In order to confirm whether the incomplete quenching was caused by the acid environment of lysosomes, co-localization analysis of **1a**-Cu (**1b**-Cu) and Lyso-Tracker Red (a commercial lysosome staining agent) was performed in HeLa cells. Firstly, 20 μM of **1a**/**1b**-Cu complexes was incubated with HeLa cells for 30 min in DMEM medium. Then the medium was replaced with fresh medium containing Lyso-Tracker Red and incubated for another 30 min. As shown in Figure 7, the bright green fluorescence of **1a**-Cu and **1b**-Cu and the red fluorescence of Lyso-Tracker Red were observed in HeLa cells. The localization of **1a**-Cu and **1b**-Cu in the cells was almost identical to that of Lyso-Tracker Red. These results suggested that **1a**-Cu and **1b**-Cu complexes can be used as fluorescence probes to specifically label lysosomes in HeLa cells.

### Characterization of 1a−1d/RNA Complexes

**Figure 8:**
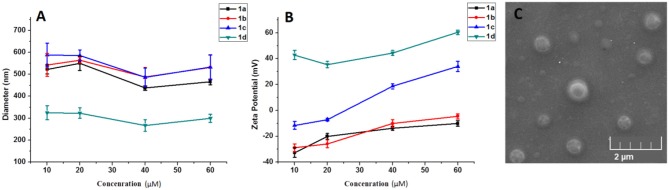
Mean particle size **(A)** and zeta-potential **(B)** of the complexes formed from the **1a**–**1d** under various concentrations (10–60 μM). Data are presented as mean ± s.d. (*n* = 3). A representative result from three independent experiments. SEM image **(C)** of **1a**/RNA complex at a concentration of 20 μM.

As a gene carrier, the proper particle size and zeta-potential are of critical importance for efficient gene delivery. Therefore, the size distribution and zeta potential of the **1a**–**1d/**RNA complexes were measured at different concentrations of MFCs **1a**–**1d** via dynamic light scattering (DLS) assay. As shown in Figure 8A, the MFCs **1a**–**1c** could efficiently condense RNA into nanoparticles with diameters ranging from 450 to 600 nm, while the particle derived from **1d**/RNA was much smaller than that of **1a−1c**/RNA complexes. It is probable that a simple long hydrophobic alkyl chain will lead to a stronger hydrophobic effect and further result in a smaller particle (Zhang et al., 2011). Meanwhile, surface potentials of **1a−1d**/RNA complexes were also measured by DLS, and the results are shown in Figure 8B. The zeta potential of the four complexes rose along with the increasing of the concentration from 10 to 60 μM. Compared to other MFCs, compound **1d** modified with octadecylamine gave higher zeta potential, indicating that the simple long hydrophobic alkyl chain would gather the positive charge. Meanwhile, the morphology and shape information of **1a**/RNA was observed by scanning electron microscopy (SEM) and is shown in Figure 8C. The MTC **1a** could compact RNA into spherical nanoparticles with a diameter of 400–600 nm at a concentration of 20 μM, which was consistent with that determined by DLS.

### Cytotoxicity of 1a−1d

**Figure 9:**
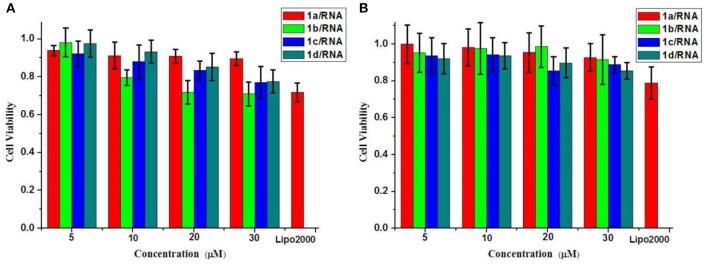
Cytotoxicities of the complexes of MFCs **1a**–**1d**/RNA at different concentrations on HeLa cells **(A)** and CCC-HPF-1 cells **(B)**. Data are presented as mean ± s.e.m. (*n* = 5).

We further evaluated the cytotoxicities of the complexes of MFCs **1a−1d**/RNA on HeLa cells and CCC-HPF-1 using a standard MTT assay. As shown in Figure 9, cytotoxicities of **1a**–**1d**/RNA showed better biocompatibility on normal cells (CCC-HPF-1) than on tumor cells (HeLa). The cell viabilities on HeL a cells were still >75% at concentrations ranging from 5 to 30 μM, which are slightly higher than that of lipofectamine 2000. Compared to complexes of **1a**–**1d**/RNA, the MFCs **1a**–**1d** (without RNA) showed higher cytotoxicity and the cell viabilities were only more than 60% in most cases (Figure S4), which should be caused by excess positive charges of triazole and [12]aneN_3_ units. It was also found that the cytotoxicity increased with the increasing concentrations of these MTCs, but it was still acceptable under the tested concentrations, indicating that it was suitable for further gene delivery.

### Cell Uptake of 1a−1d/RNA Complexes

**Figure 10:**
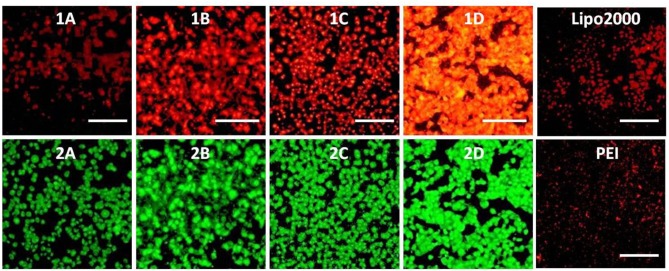
Fluorescence microscope images of HeLa cells transfected with Cy5-labeled RNA (9 μg/mL) by MFCs **1a**–**1d** at a concentration of 20 μM, 25 kD PEI, and lipofectamiine 2000 as positive control. **(1A–D)**: red channels, **(2A–D)**: green channels. A representative result from three independent experiments. The scale bar in the figure is 40 μm.

To investigate the capacity of these MTCs to delivery RNA as non-viral vectors, cellular uptake mediated by **1a**–**1d** was carried out by using fluorescence microscopy. Twenty-five kilodaltons of PEI and lipofectamine 2000 served as positive controls. In order to observe the information of cellular uptake, siRNA was labeled with a red dye Cy5. Before a direct comparison on RNA delivery ability of these materials, cellular uptake mediated by **1c** was firstly performed at different concentrations. The w/w ratios of PEI/RNA and lipofectamine 2000/RNA were also screened on the HeLa cells. It was found that the cellular uptake mediated by **1c** showed the highest red fluorescence density at the concentration of 20 μM (Figure S5). Meanwhile, the best w/w ratios for PEI/RNA and lipofectamine 2000/RNA were 5/1 and 20/1, respectively (Figures S6, S7). Subsequently, RNA delivery mediated by **1a**–**1d** was carried out at a concentration of 20 μM in HeLa cells. As shown in Figure 10, all the MTCs exhibited good cellular uptake ability, and almost all the cells were full of green fluorescence. Among these MTCs, compound **1d** modified with octadecylamine gave the strongest intensity of red fluorescence, indicating the best RNA delivery ability, which is much better than that of lipofectamine 2000 and PEI.

**Figure 11:**
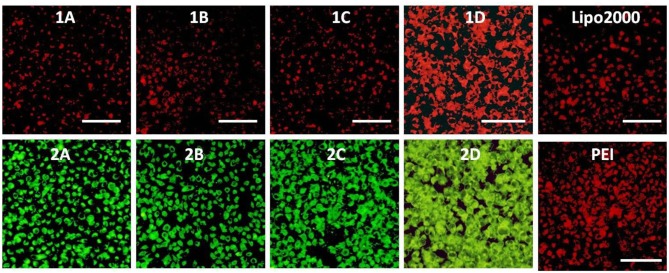
Fluorescence microscope images of HepG2 cells transfected with Cy5-labeled RNA (9 μg/mL) by MFCs **1a**–**1d** at a concentration of 20 μM, 25 kD PEI and lipofectamiine 2000 as positive control. **(1A–D)**: red channels, **(2A–D)**: green channels. A representative result from three independent experiments. The scale bar in the figure is 40 μm.

**Figure 12:**
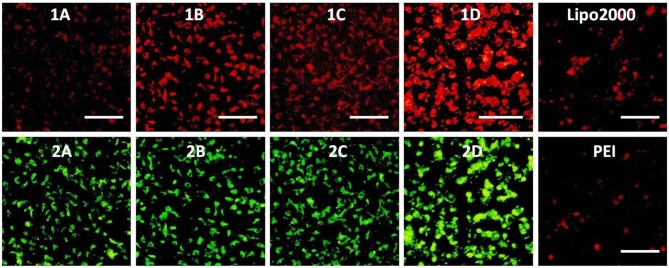
Fluorescence microscope images of U2O cells transfected with Cy5-labeled RNA (9 μg/mL) by MFCs **1a**–**1d** at a concentration of 20 μM, 25 kD PEI and lipofectamiine 2000 as positive control. **(1A–D)**: red channels, **(2A–D)**: green channels. A representative result from three independent experiments. The scale bar in the figure is 40 μm.

**Figure 13:**
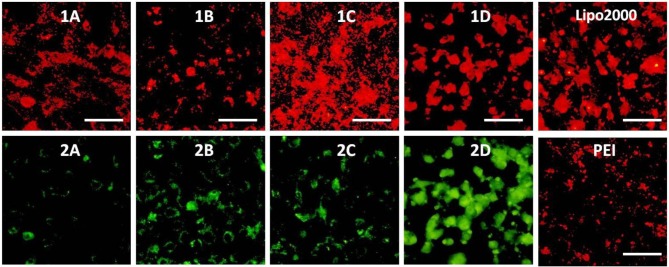
Fluorescence microscope images of MC3T3-E1 cells transfected with Cy5-labeled RNA (9 μg/mL) by MFCs **1a**–**1d** at a concentration of 20 μM, 25 kD PEI and lipofectamiine 2000 as positive control. **(1A–D)**: red channels, **(2A–D)**: green channels. A representative result from three independent experiments. The scale bar in the figure is 50 μm.

The RNA delivery ability of **1a**–**1d** was further evaluated in other cell lines. As shown in Figures 11–13, the RNA delivery ability of **1d** was superior to those of **1a**, **1b**, and **1c** in HepG2, U2Os, and MC3T3-E1 cells, suggesting that simple long hydrophobic chain-modified MTC could enhance cellar uptake ability. However, when an ester bond was introduced to the long hydrophobic chain (**1c**), cellar uptake ability would decrease (**1c**<**1d**). MTC **1d** exhibited excellent RNA delivery ability in the above three cell lines, and its performance was superior to lipofectamine 2000 and PEI, which could be ascribed to the small size of the nanoparticle and high surface potential of **1d**/RNA complex, indicating its potential application as a non-viral vector.

## Conclusion

A series of 1,8-naphthalimide-modified MFCs **1a**–**1d** were synthesized. They exhibited excellent recognition ability for Cu^2+^ in aqueous solutions and organic solvents. Due to the good water solubility and high selectivity, MFCs **1a**–**1d** were successfully applied to detect Cu^2+^ in real-time imaging in living HeLa cells. Furthermore, due to high sensitivity toward pH value ranging from 4.5 to 5.5, **1a**-Cu and **1b**-Cu were successfully used as fluorescence probes to specifically stain lysosomes in HeLa cells. The RNA delivery ability of MTCs **1a**–**1d** was also evaluated by cellular uptake experiments in HeLa, HepG2, U2Os, and MC3T3-E1 cells. They all exhibited good performance on cellular uptake, especially for MTC **1d**, the greatest red fluorescence observed in HeLa cells, which was much better than that of lipofectamine 2000 and PEI. These results will give us further insight to design high-performance multifunctional compounds for fluorescence probes and non-viral vectors.

## Data Availability

All datasets generated for this study are included in the manuscript/Supplementary Files.

## Author Contributions

Y-GG and F-LL writing-original draft preparation. SP, D-JL, AQ, XL, YT, and YL writing-review and editing. A-RQ funding acquisition.

### Conflict of Interest Statement

The authors declare that the research was conducted in the absence of any commercial or financial relationships that could be construed as a potential conflict of interest.
