# Supplementary material for: 1,8-Naphthalimide-Based Multifunctional Compounds as Cu2+ Probes, Lysosome Staining Agents, and Non-viral Vectors
Source: Front Chem. 2019 Sep 10;7:616. doi: 10.3389/fchem.2019.00616 (PMC6747039; doi:10.3389/fchem.2019.00616)
Supplement: Supplementary file 1 [file Table_1.docx]

**Electronic Supplementary Information (ESI)**

**1,8-Naphthalimide based multifunctional compounds as Cu^2+^ probes, lysosome staining agents and non-viral vectors**

Yong-Guang Gao^abc^, Fen-Li Liu^abc^, Suryaji Patil^abc^, Di-Jie Li^abc^, Abdul Qadir^abc^, Xiao Lin^abc^, Ye Tian^abc^, Yu Li^abc^ and Ai-Rong Qian^abc*^

^a^ Lab for Bone Metabolism, Key Lab for Space Biosciences and Biotechnology, School of Life Sciences, Northwestern Polytechnical University, Xi’an, Shaanxi 710072, China

^b^ Research Center for Special Medicine and Health Systems Engineering, School of Life Sciences, Northwestern Polytechnical University，Xi’an, Shaanxi 710072, China

^c^ NPU-UAB Joint Laboratory for Bone Metabolism, School of Life Sciences, Northwestern Polytechnical University，Xi’an, Shaanxi 710072, China

** Corresponding authors, Tel.: +86-29-88491840,* [*qianair@nwpu.edu.cn*](mailto:qianair@nwpu.edu.cn) *(A.-R. Qian).*

**Contents**

**1. Spectroscopic properties of 1a-1d**

**2. Characterization of 1a-1d/RNA complexes**

**3. Spectra**

**1. Spectroscopic properties of 1a-1d**


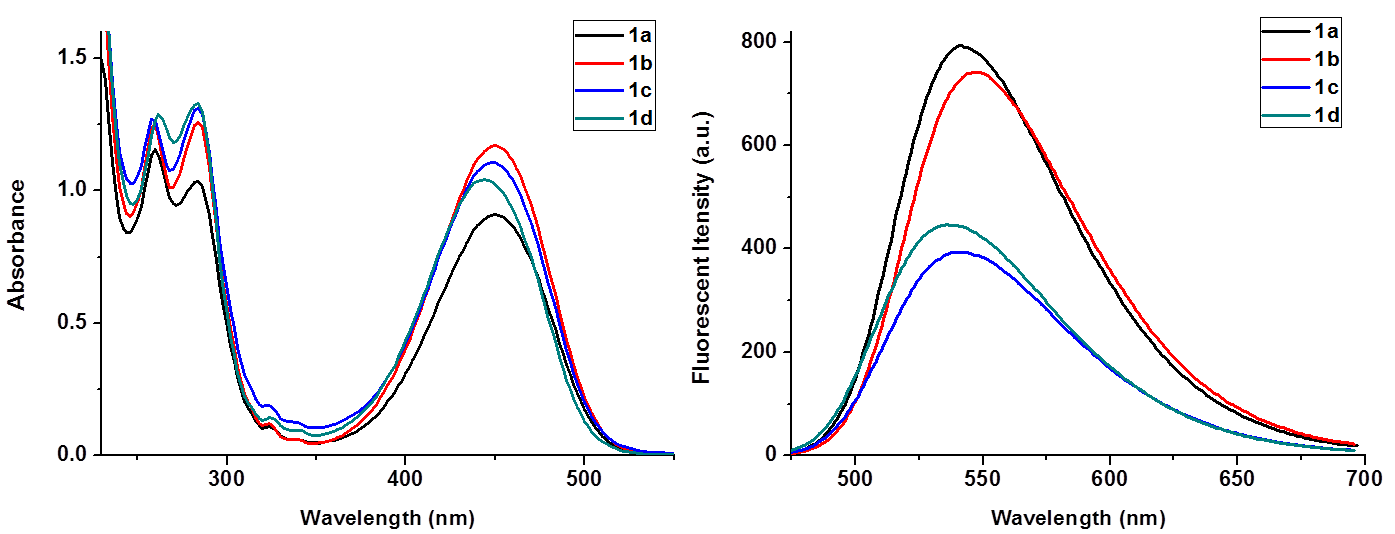


**Fig. S1** The absorption spectra (50 μM) and fluorescence (10 μM) spectra of **1a-1d** in water-Tris-HCl buffer (1 mM, pH = 7.2).


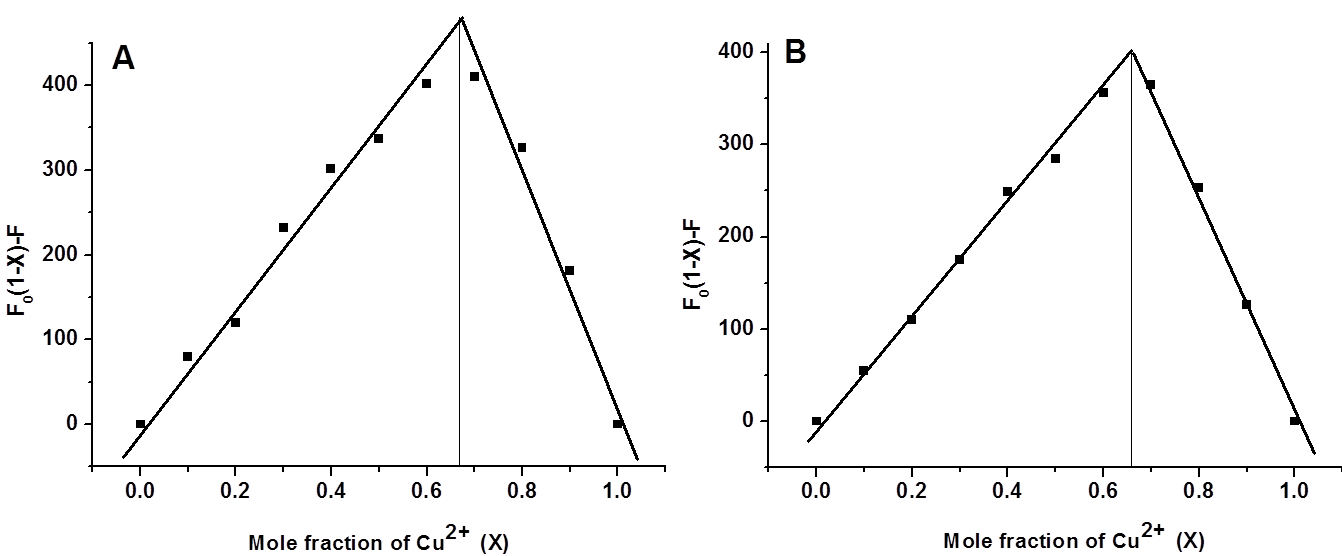

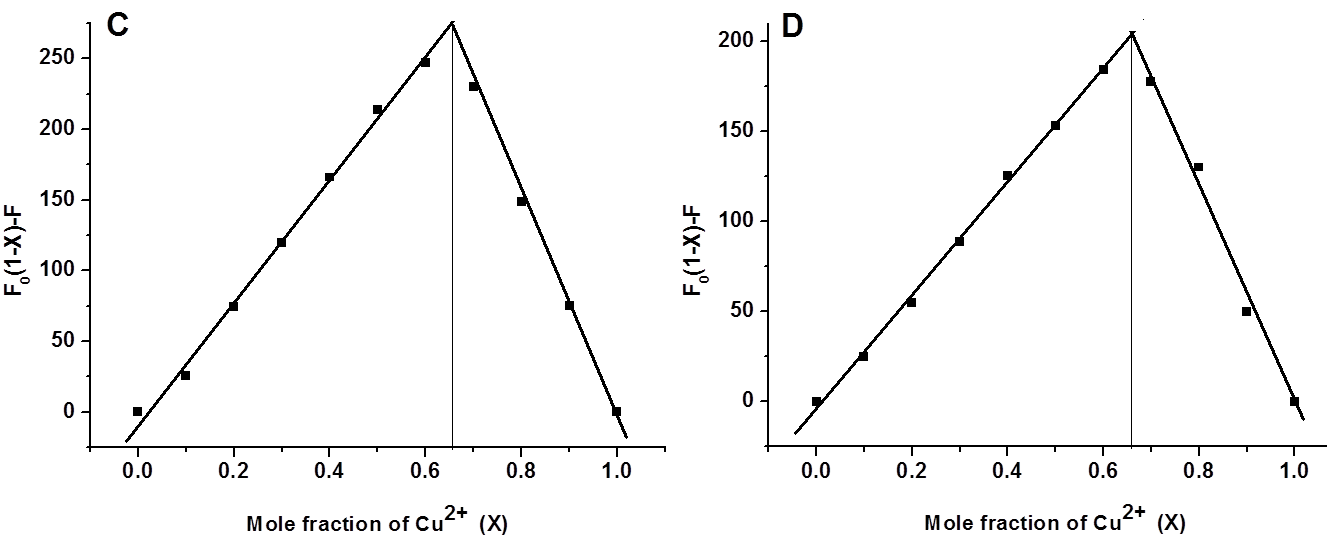


**Fig. S2** Job's plot showing the 1:1 (**1a**-**1d**/Cu^2+^) complex stiochiometry, mole fraction of Cu^2+^ X = [Cu^2+^]/([Cu^2+^]+[**1**]), [Cu^2+^]+[**1**] = 30 μM in Tris-HCl buffer.

**Fig. S3** The proposed binding modes of **1** and Cu^2+^.

**Fig. S4** Cytotoxicities of the compounds **1a**-**1d** at different concentrations on HeLa cells

**2. Cell uptake of 1a-1d/RNA complexes**


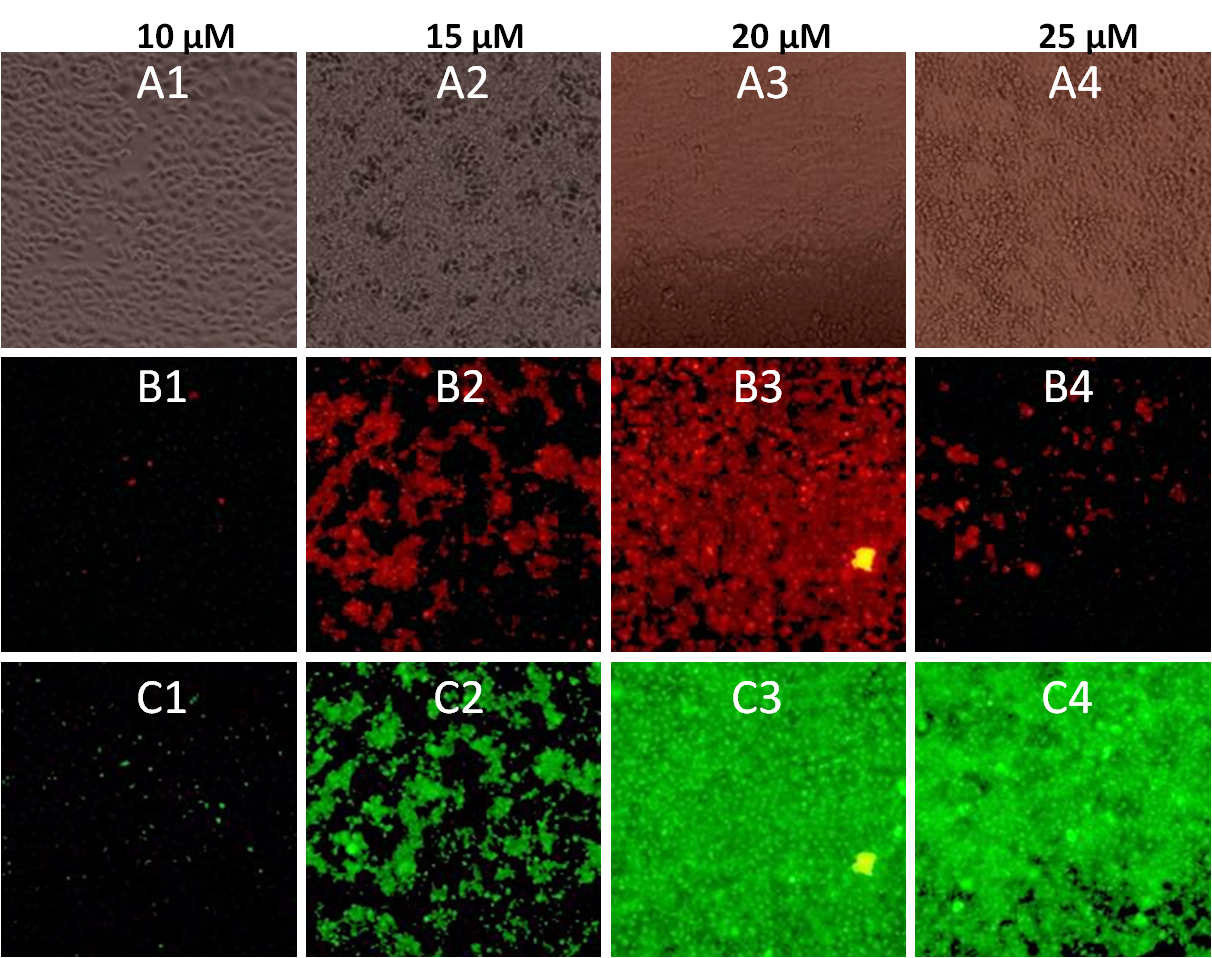


**Fig. S5** Fluorescence microscope images of HeLa cells transfected with Cy5-labaled siRNA (9 μg/mL) by **1c** at different concentrations (10 μM, 15 μM, 20 μM, 25 μM). A1-A4: BF, B1-B4: red channels, C1-C4: green channels.


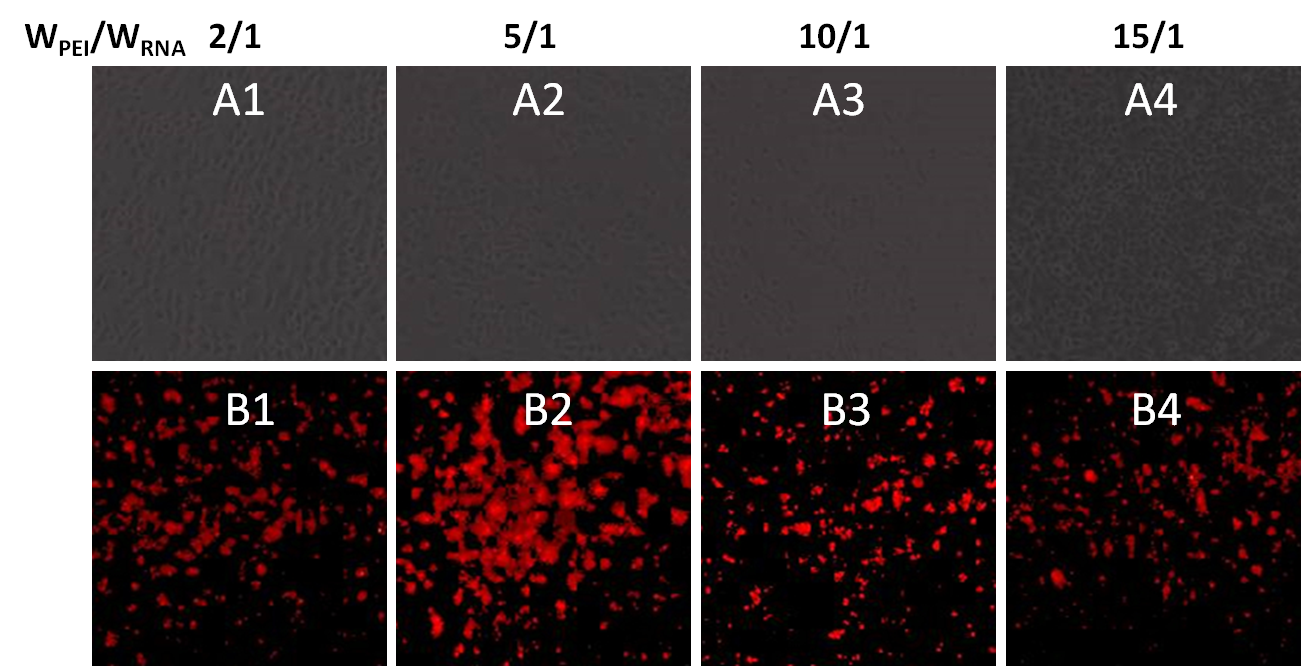


**Fig. S6** Fluorescence microscope images of HeLa cells transfected with Cy5-labaled siRNA (9 μg/mL) by 25 KDa PEI at different weight ratios. A1-A4: BF, B1-B4: red channels**.**


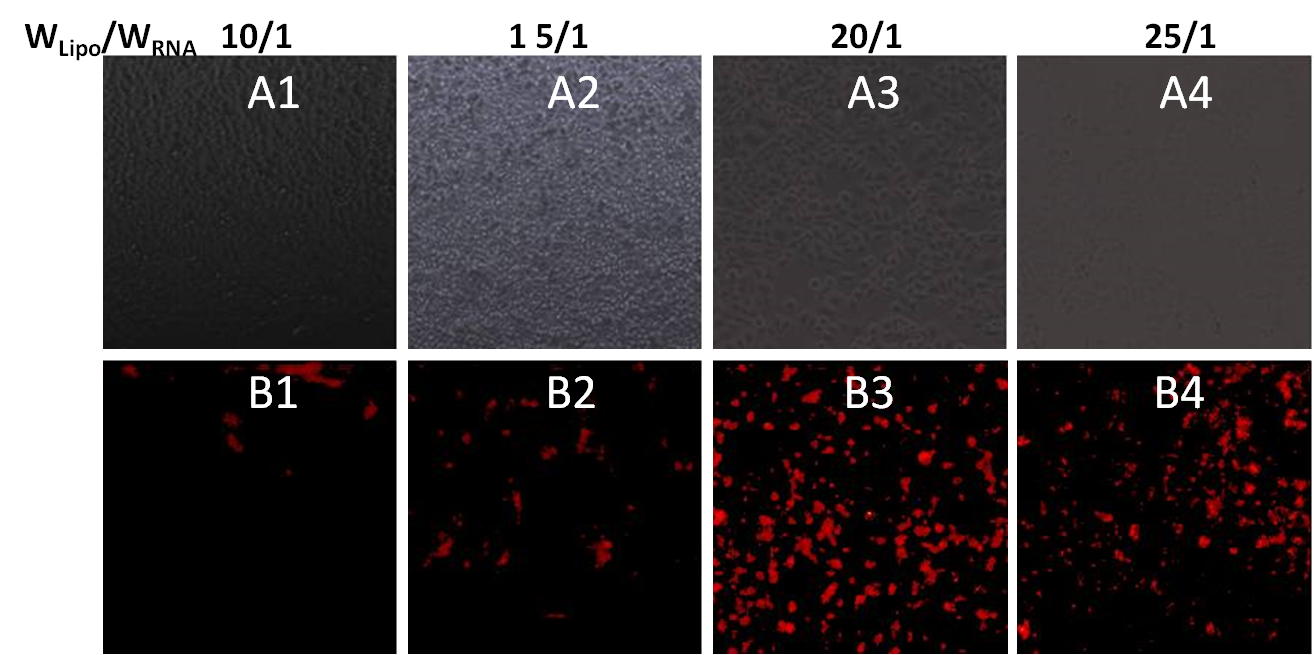


**Fig. S7** Fluorescence microscope images of HeLa cells transfected with Cy5-labaled siRNA (9 μg/mL) by lipofectamine 2000 at different weight ratios. A1-A4: BF, B1-B4: red channels.

**3 Spectra**


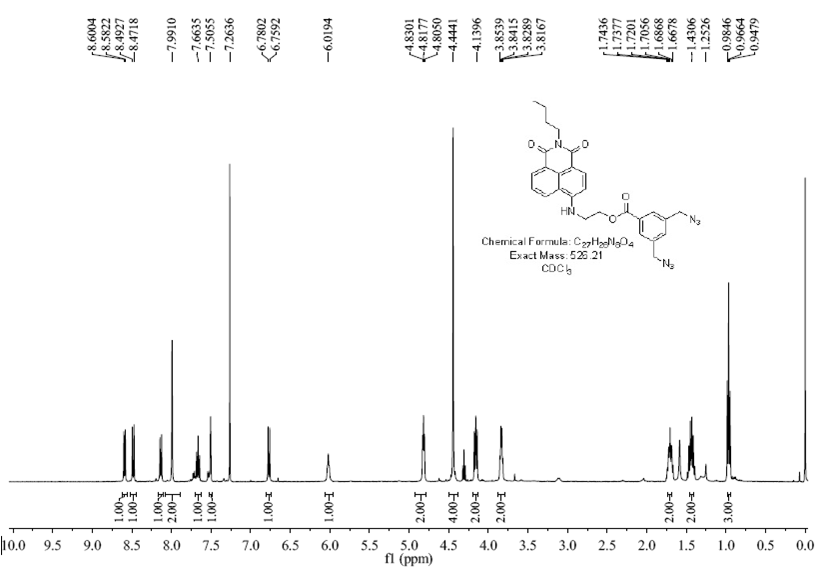


^1^H NMR spectrum of compound **4a** (solvent: CDCl_3_)


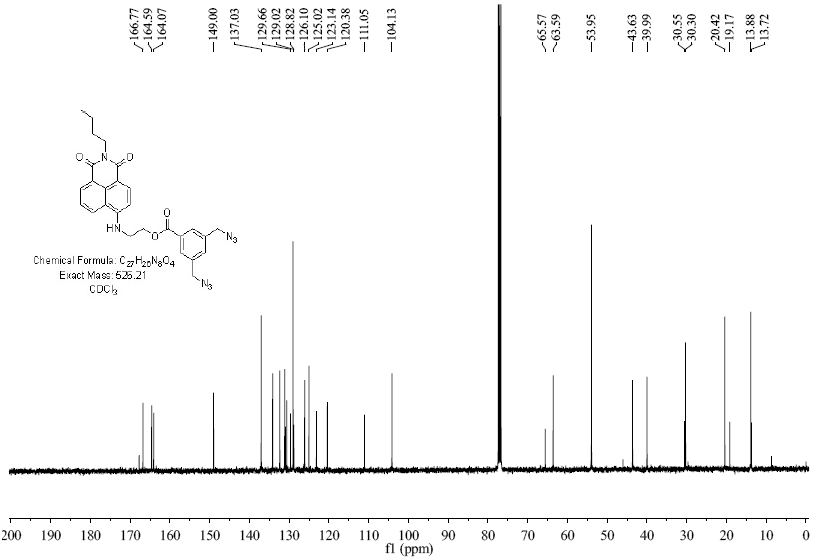


^13^C NMR spectrum of compound **4a** (solvent: CDCl_3_)


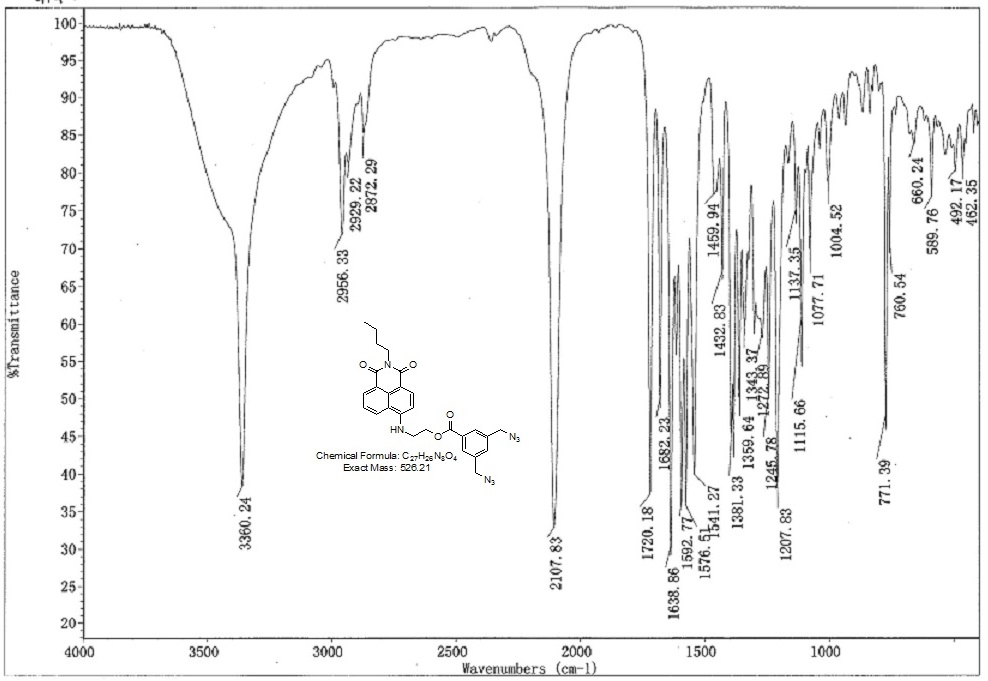


IR spectrum of compound **4a**


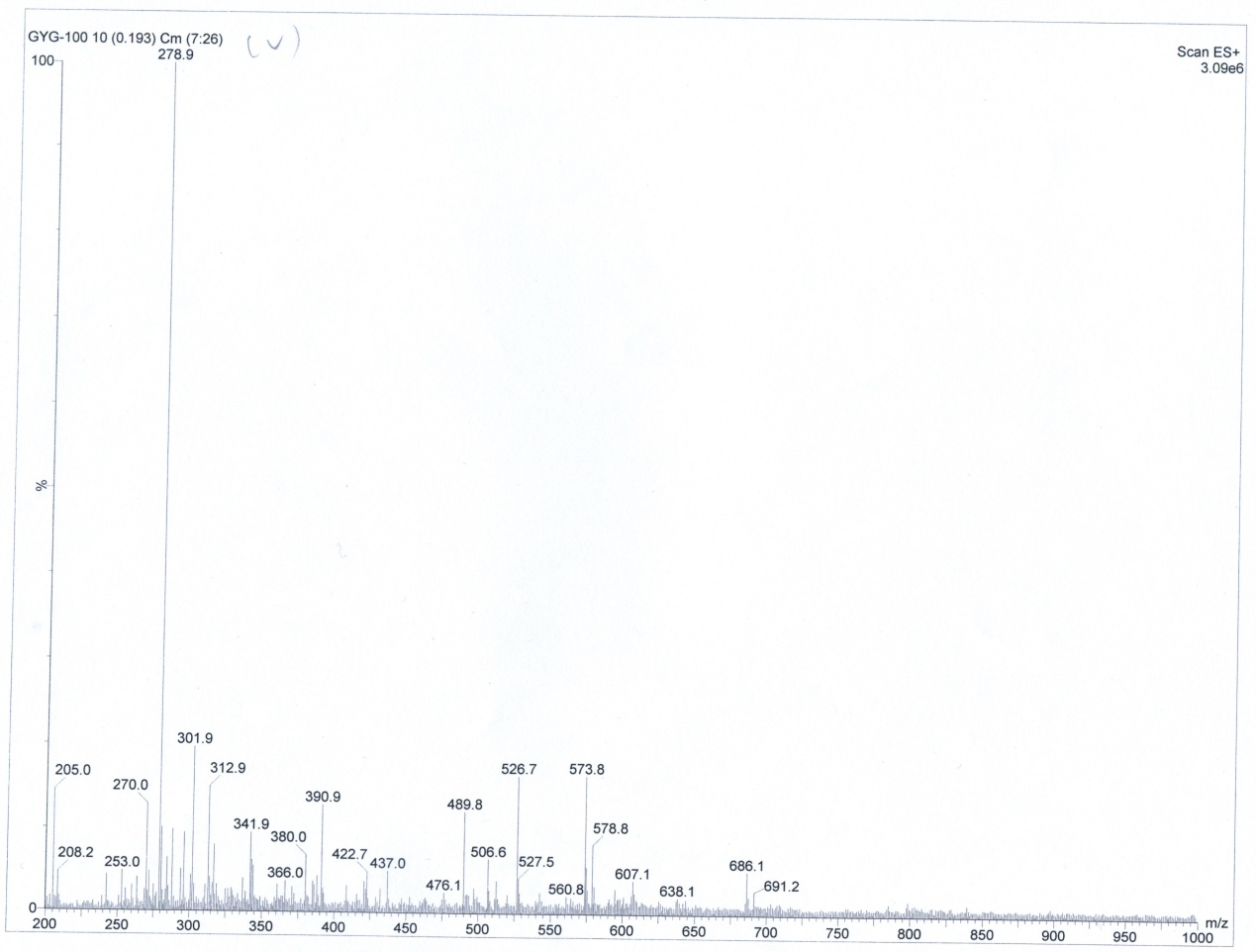


Ms spectrum of compound **4a**


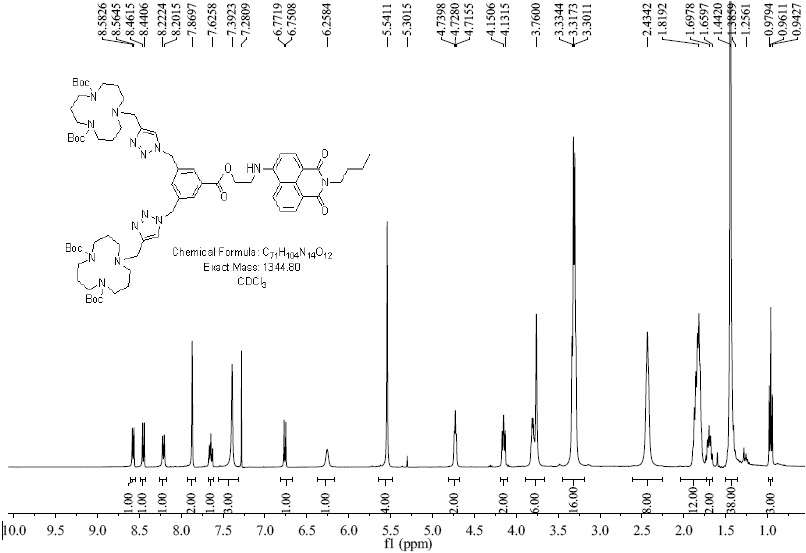


^1^H NMR spectrum of compound **6a** (solvent: CDCl_3_)


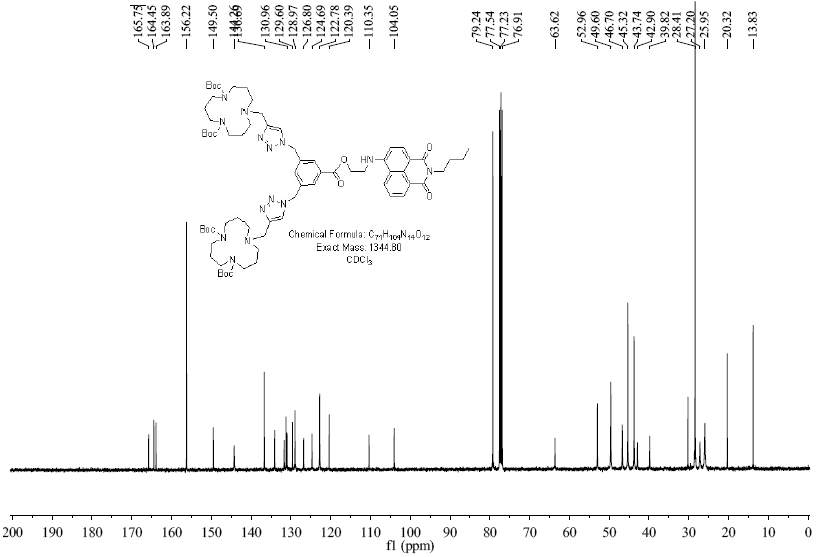


^13^C NMR spectrum of compound **6a** (solvent: CDCl_3_)


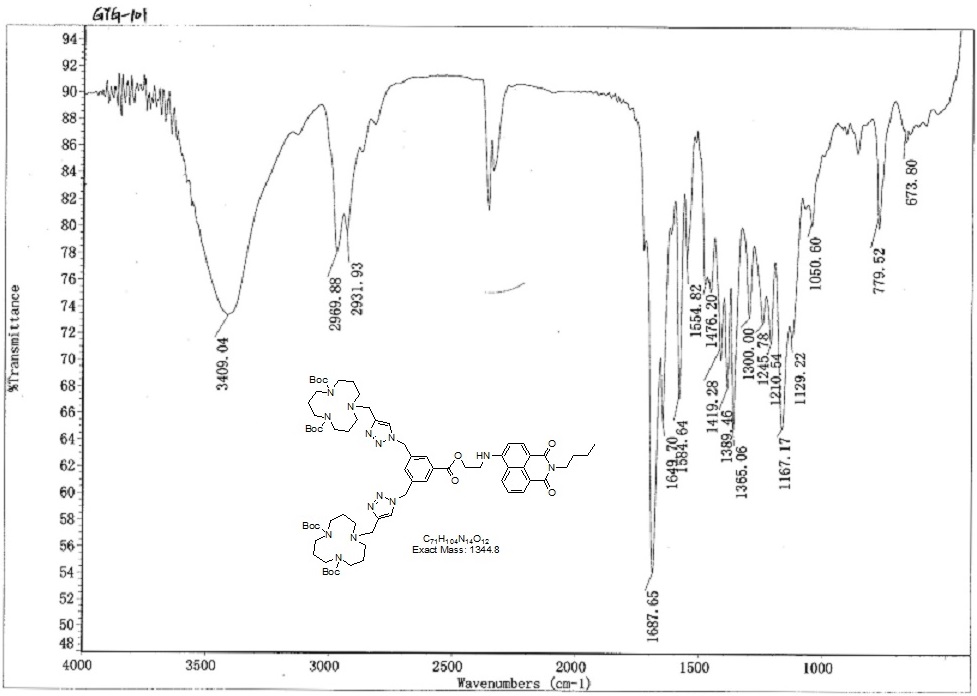


IR spectrum of compound **6a**


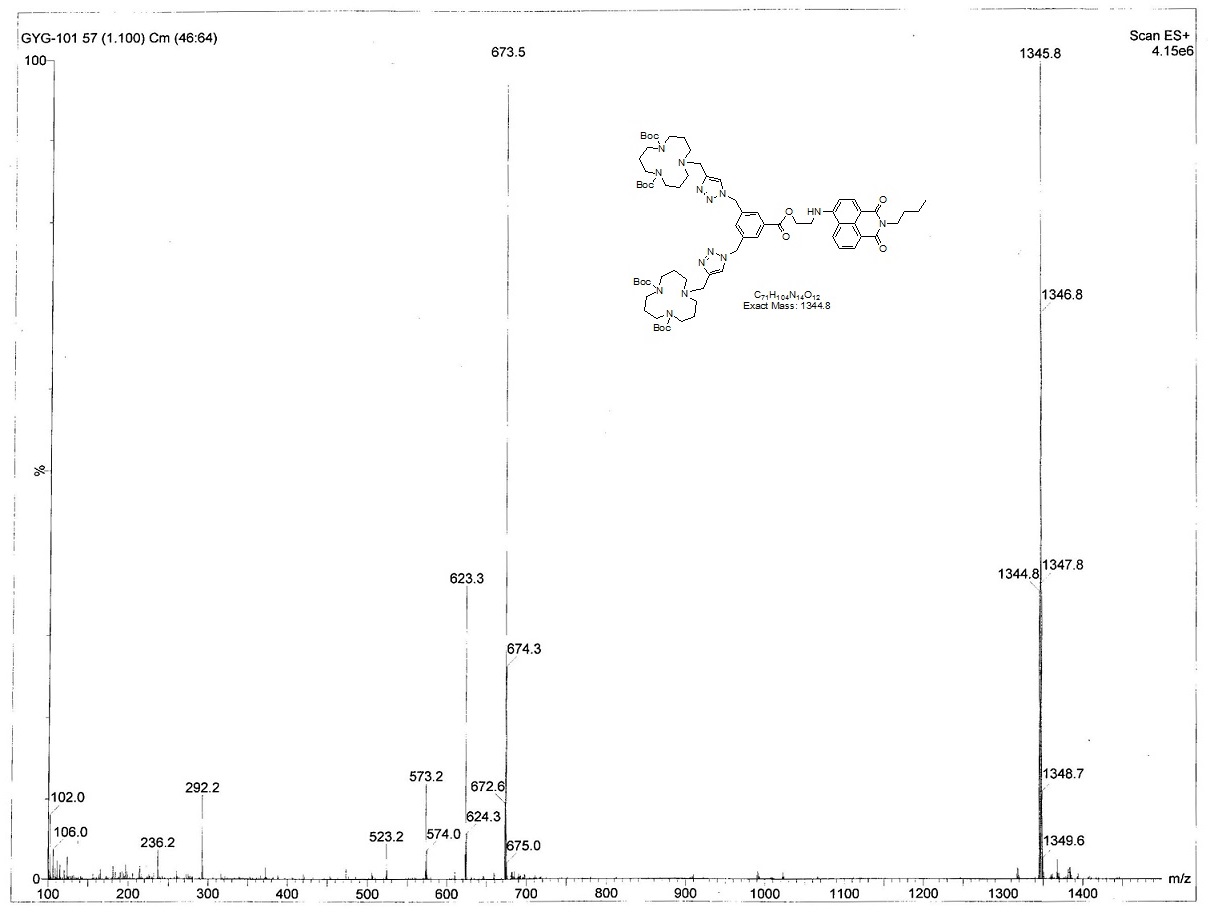


Ms spectrum of compound **6a**


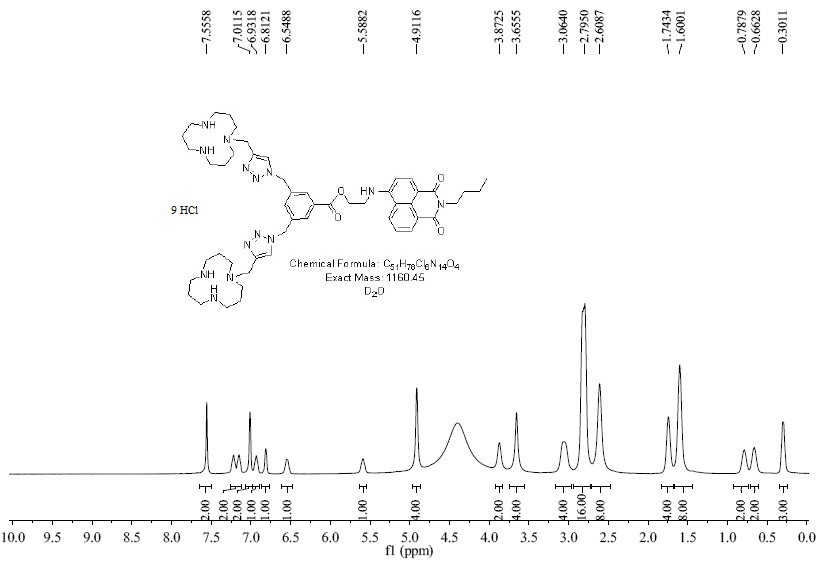


^1^H NMR spectrum of compound **1a** (solvent: D_2_O)


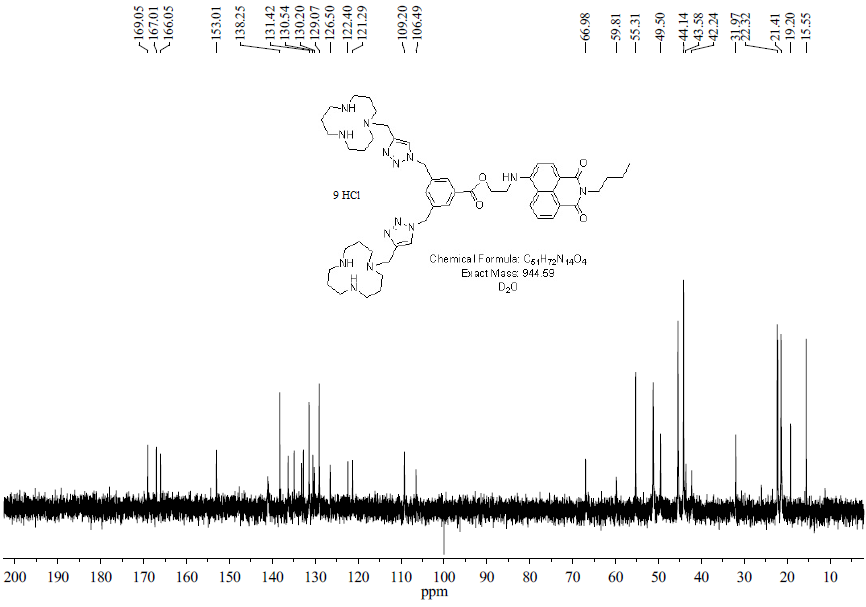


^13^C NMR spectrum of compound **1a** (solvent: D_2_O)


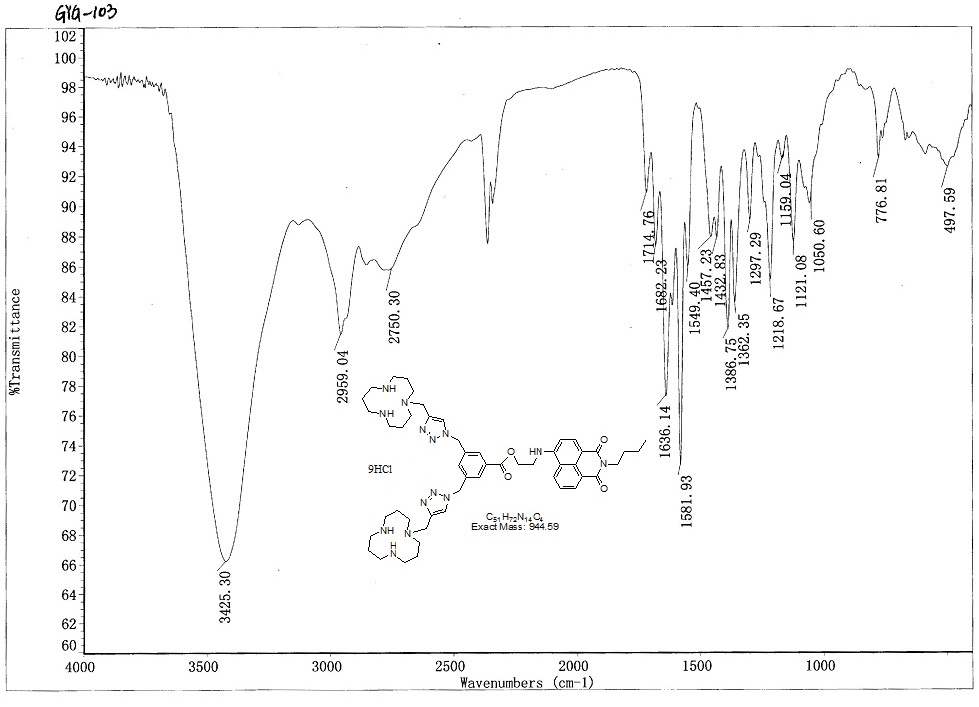


IR spectrum of compound **1a**


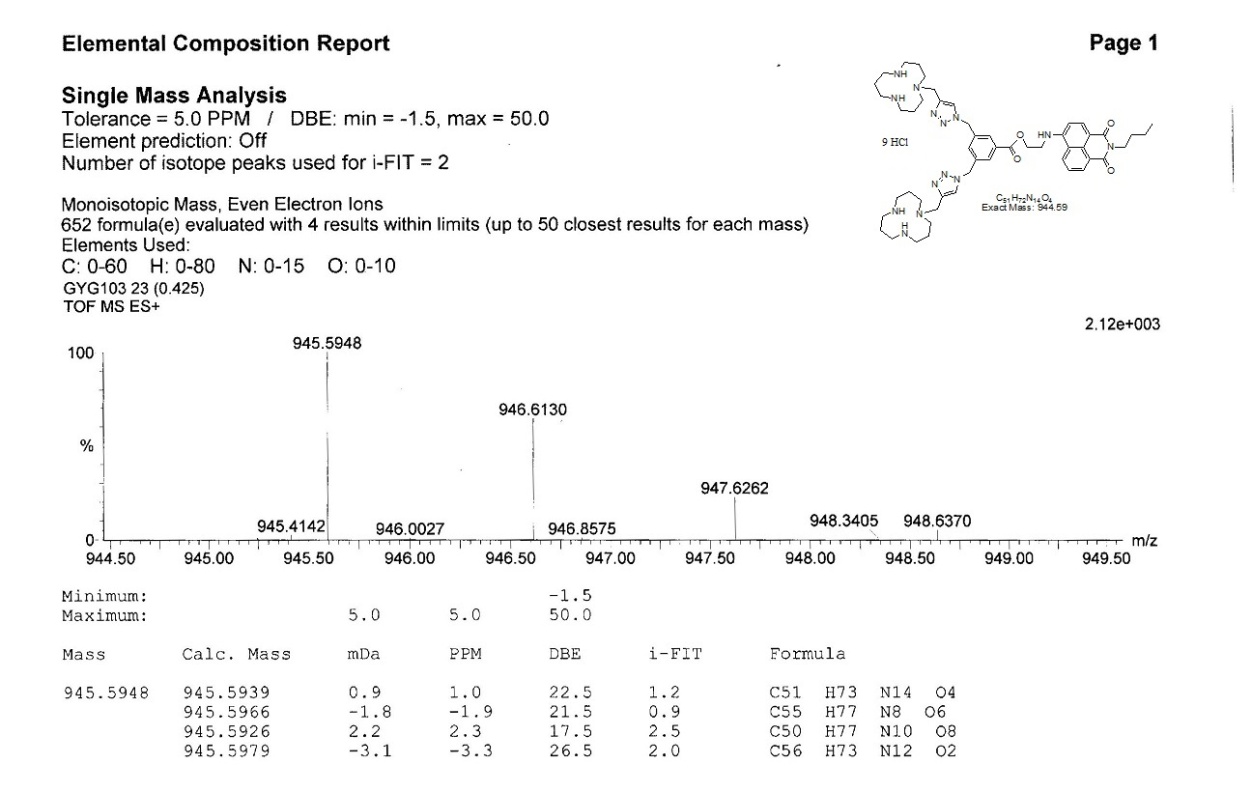
HR-Ms spectrum of compound **1a**


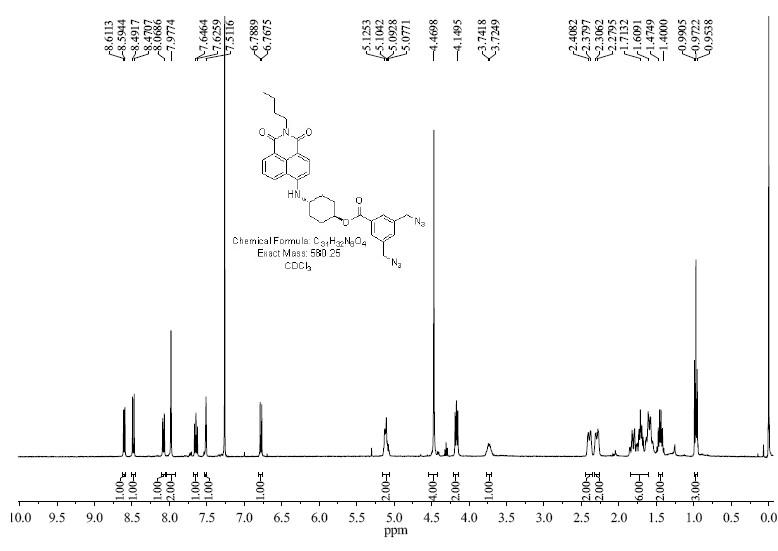


^1^H NMR spectrum of compound **4b** (solvent: CDCl_3_)


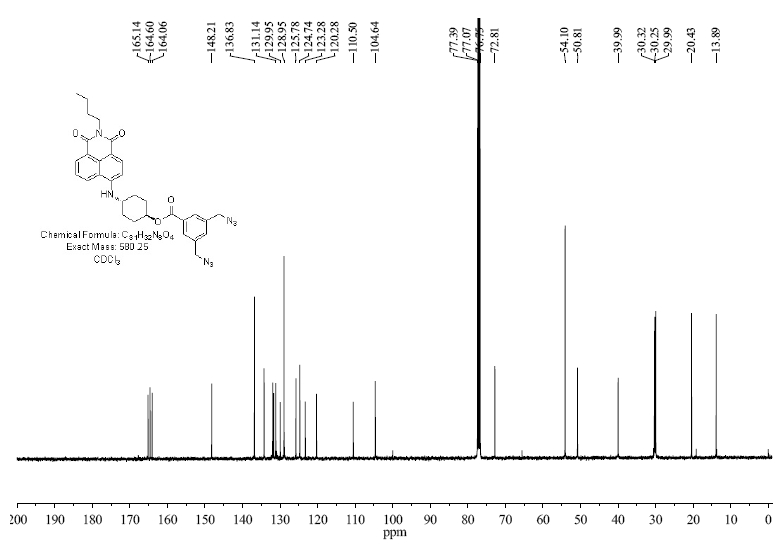


^13^C NMR spectrum of compound **4b** (solvent: CDCl_3_)


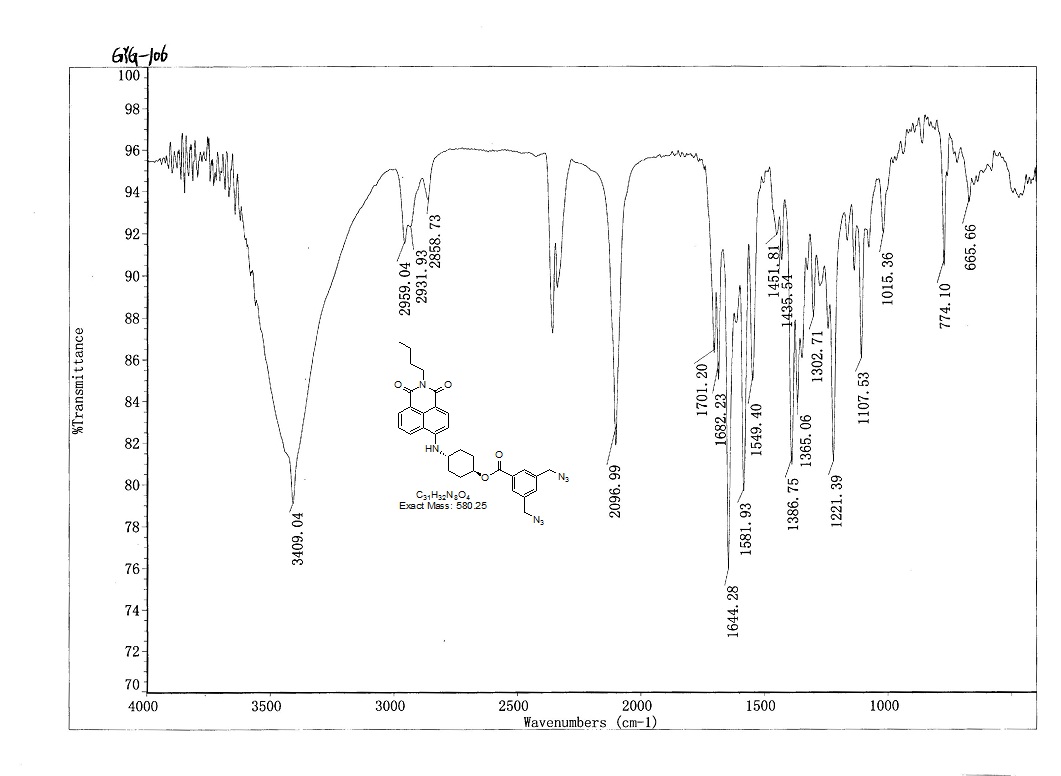


IR spectrum of compound **4b**


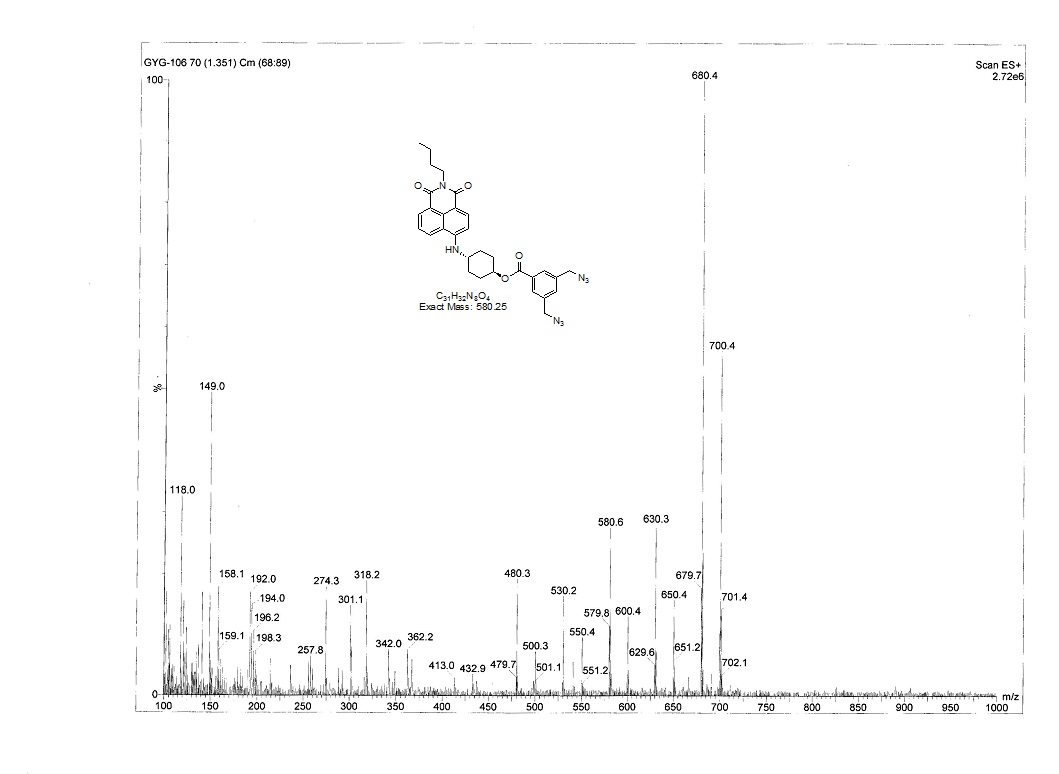


Ms spectrum of compound **4b**


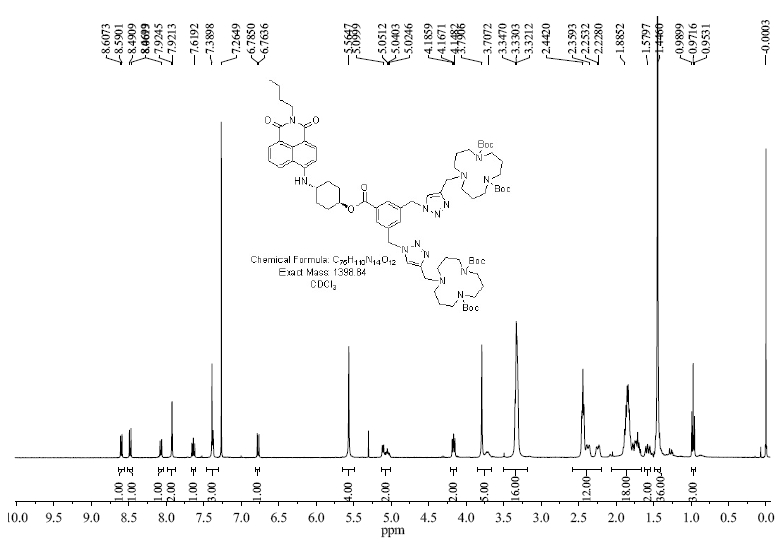


^1^H NMR spectrum of compound **6b** (solvent: CDCl_3_)


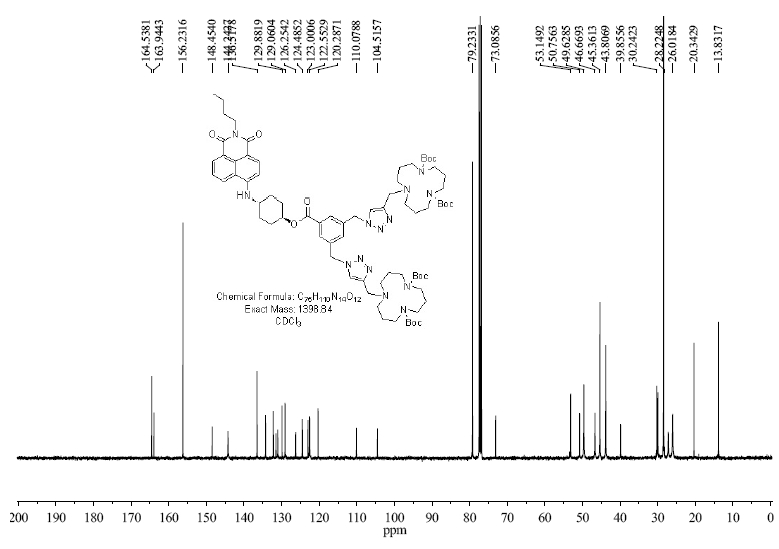


^13^C NMR spectrum of compound **6b** (solvent: CDCl_3_)


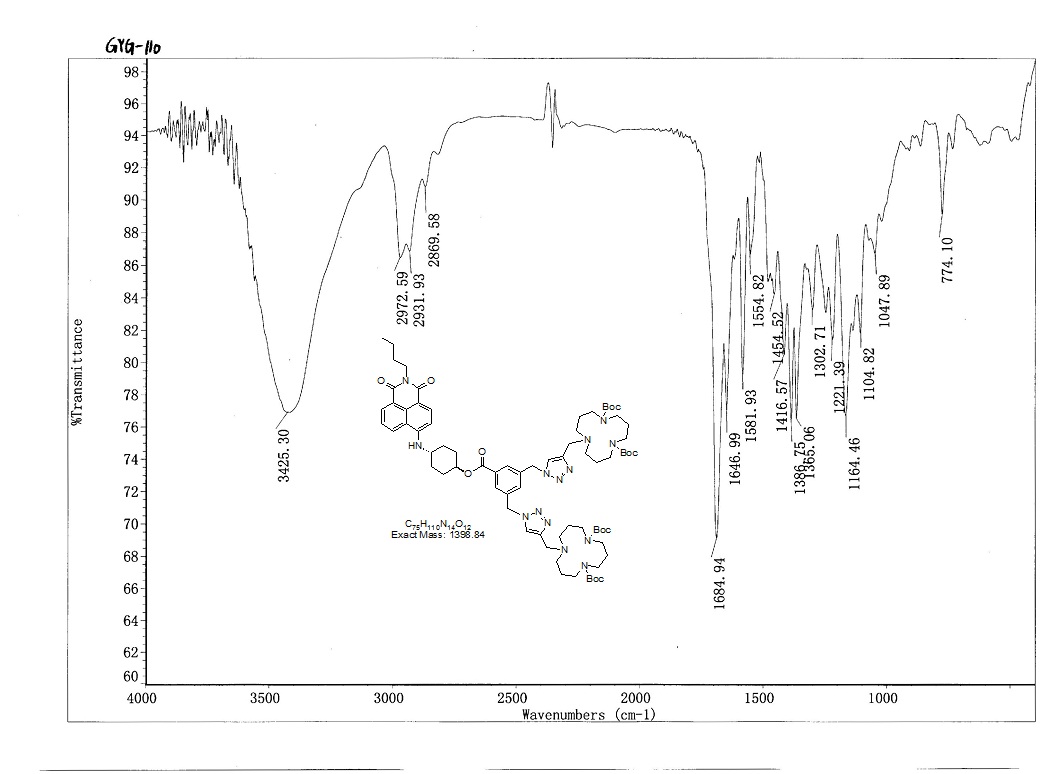


IR spectrum of compound **6b**


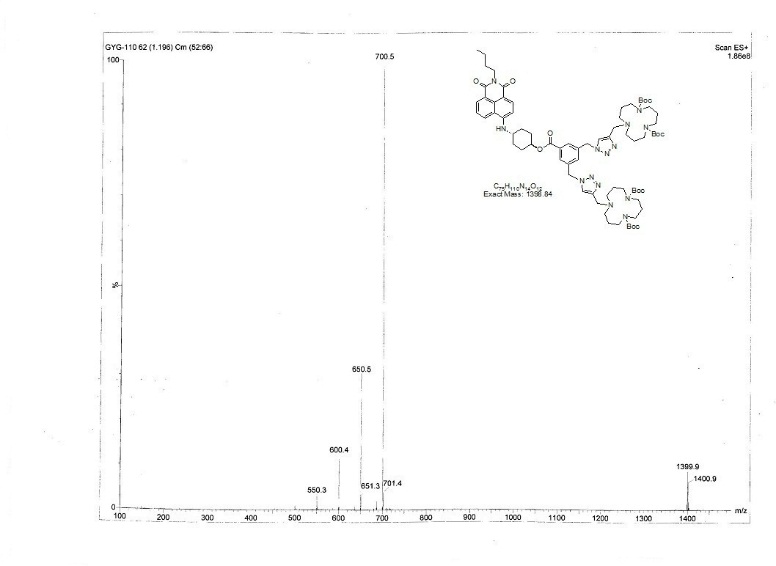


Ms spectrum of compound **6b**


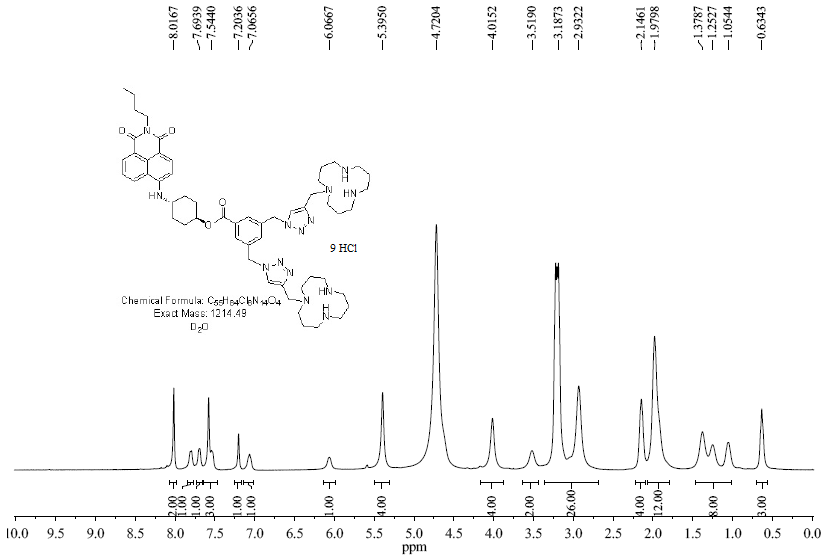


^1^H NMR spectrum of compound **1b** (solvent: D_2_O)


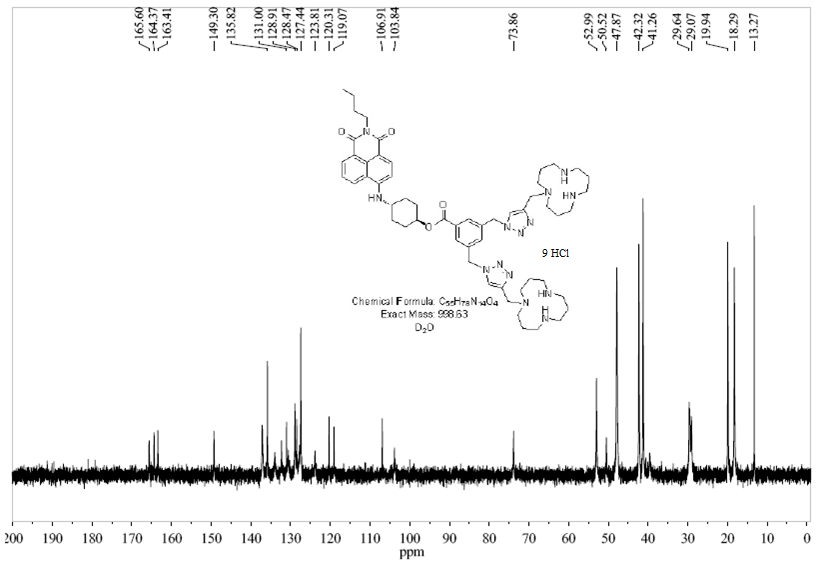


^13^C NMR spectrum of compound **1b** (solvent: D_2_O)


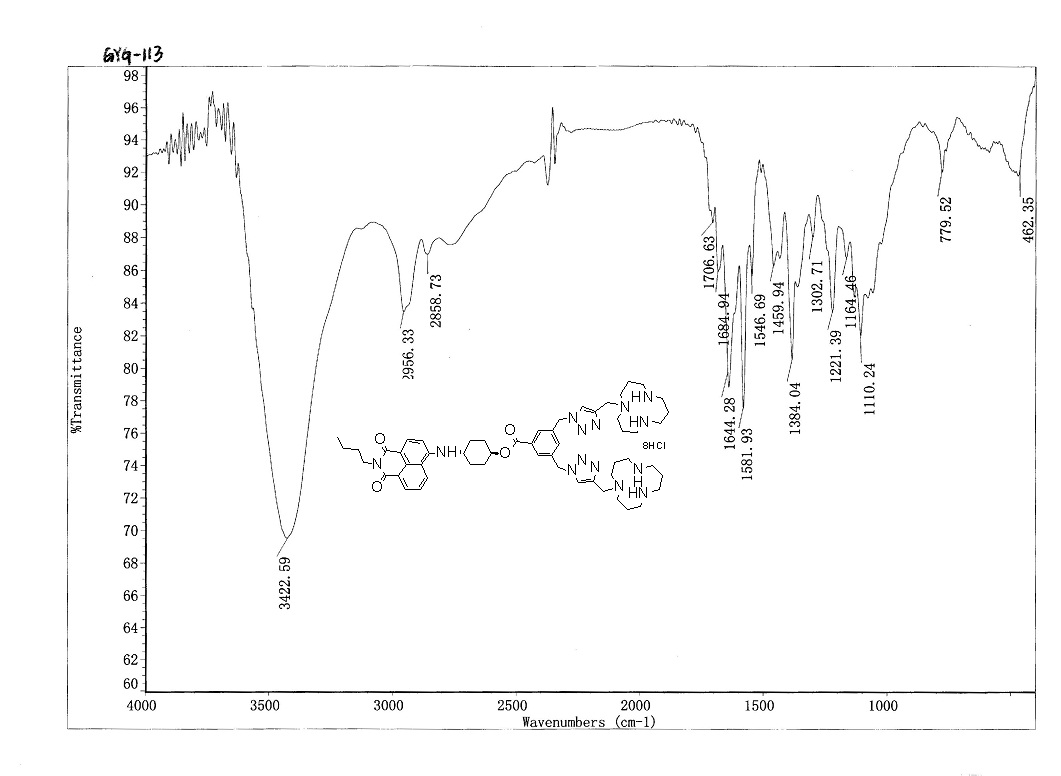


IR spectrum of compound **1b**


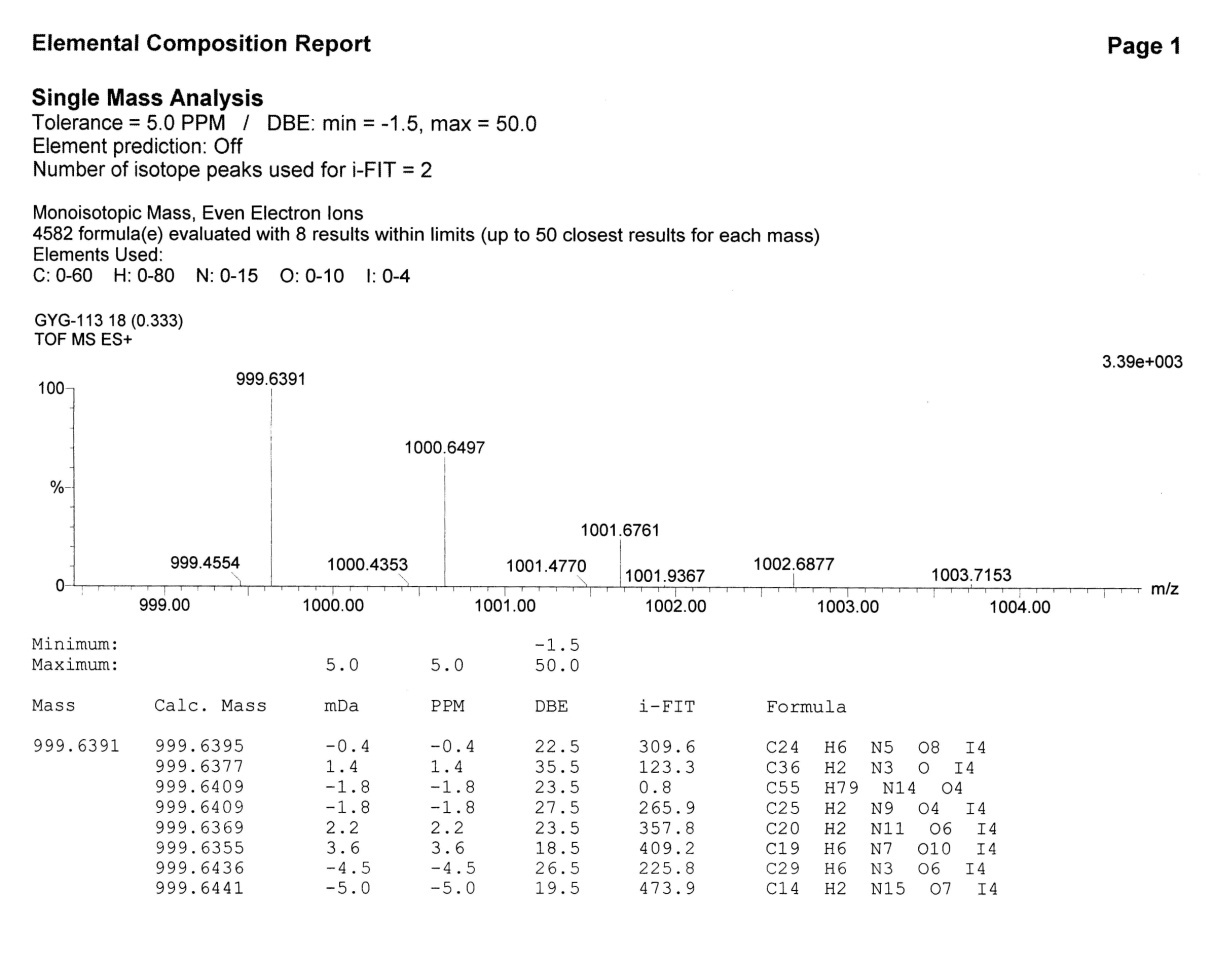


HR-Ms spectrum of compound **1b**
